# Associations between modifiable risk factors and white matter of the aging brain: insights from diffusion tensor imaging studies

**DOI:** 10.1016/j.neurobiolaging.2019.04.006

**Published:** 2019-08

**Authors:** Thomas M. Wassenaar, Kristine Yaffe, Ysbrand D. van der Werf, Claire E. Sexton

**Affiliations:** a Nuffield Department of Clinical Neurosciences, Wellcome Centre for Integrative Neuroscience, FMRIB Centre, University of Oxford, John Radcliffe Hospital, UK; b Departments of Psychiatry, Neurology, and Epidemiology and Biostatistics, University of California San Francisco, San Francisco, CA, USA; c Department of Anatomy and Neurosciences, VU University Medical Center, MC, Amsterdam, the Netherlands; d Department of Neurology, Global Brain Health Institute, Memory and Aging Center, University of California San Francisco, San Francisco, CA, USA; e Department of Psychiatry, Wellcome Centre for Integrative Neuroscience, Oxford Centre for Human Brain Activity, University of Oxford, John Radcliffe Hospital, UK

**Keywords:** Aging, Diffusion tensor imaging, White matter, Modifiable– risk factor

## Abstract

There is increasing interest in factors that may modulate white matter (WM) breakdown and, consequentially, age-related cognitive and behavioral deficits. Recent diffusion tensor imaging studies have examined the relationship of such factors with WM microstructure. This review summarizes the evidence regarding the relationship between WM microstructure and recognized modifiable factors, including hearing loss, hypertension, diabetes, obesity, smoking, depressive symptoms, physical (in) activity, and social isolation, as well as sleep disturbances, diet, cognitive training, and meditation. Current cross-sectional evidence suggests a clear link between loss of WM integrity (lower fractional anisotropy and higher mean diffusivity) and hypertension, obesity, diabetes, and smoking; a relationship that seems to hold for hearing loss, social isolation, depressive symptoms, and sleep disturbances. Physical activity, cognitive training, diet, and meditation, on the other hand, may protect WM with aging. Preliminary evidence from cross-sectional studies of treated risk factors suggests that modification of factors could slow down negative effects on WM microstructure. Careful intervention studies are needed for this literature to contribute to public health initiatives going forward.

## Highlights

• Both aging and dementia are associated with breakdown of white matter (WM) microstructure.
• We review a range of modifiable factors that could prevent or slow age-related WM decline.
• Risk factors are consistently related with lower fractional anisotropy and higher mean diffusivity.
• Treatment of risk factors may slow WM decline.
• Careful longitudinal and intervention studies are now needed.

## 1 Introduction

**Fig. 1:**
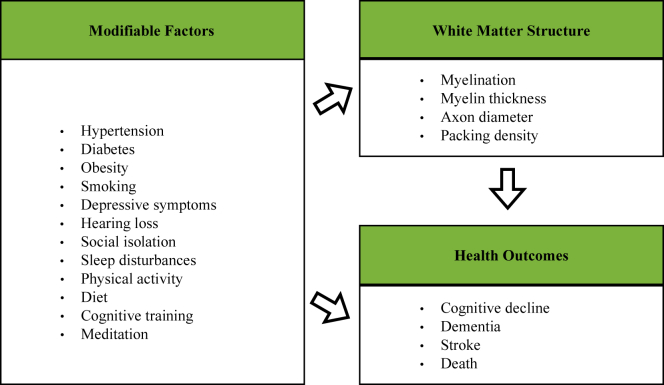
Modifiable factors linked to adverse health outcomes. A range of modifiable factors have been linked to adverse health outcomes in aging, including cognitive decline and dementia (Debette and Markus, 2010, Prins and Scheltens, 2015). A substantial body of work has implicated the brain in this pathway. For years, researchers have focused on the mediating role of gray matter in the relationship between lifestyle factors and health outcomes. However, it has become increasingly clear that white matter structure is an important mediator too. For instance, white matter macrostructural alterations, such as hyperintensities, are well-known to increase risk for dementia and stroke (Debette and Markus, 2010). (For interpretation of the references to color in this figure legend, the reader is referred to the Web version of this article.)

White matter (WM) pathways play an essential role in the human brain by connecting distributed regions and enabling efficient exchange of information. WM is crucial for efficient cognitive functioning (Filley and Fields, 2016), and changes in its make-up shape human behavior and underlie learning (Zatorre et al., 2012). During the aging process, WM loss outpaces the loss of gray matter (Guttmann et al., 1998), and decline in WM integrity is associated with cognitive decline and variation in performance (Bennett and Madden, 2014, Walhovd et al., 2014). WM decline further increases risk of various brain disorders (Fig. 1) and is a feature of several dementias (Debette and Markus, 2010, Filley and Fields, 2016, Prins and Scheltens, 2015). In Alzheimer's disease (AD), for instance, WM abnormalities have consistently been implicated in its pathogenesis and are now considered a core feature of AD (Nasrabady et al., 2018). WM disruption, including myelin loss and oligodendrocyte dysfunction, may be among the earliest pathological changes in AD, has been related to AD-related cognitive deficits, and could be considered as a target for early treatment (Nasrabady et al., 2018). Encouragingly, WM of the adult brain demonstrates plasticity, with structural changes including myelin formation and remodeling occurring over hours, days, weeks, and months (Sampaio-Baptista and Johansen-Berg, 2017).

Over the past decades, the scientific community has become increasingly interested in lifestyle factors that may modulate WM decline, in attempts to slow, delay, or prevent age-related cognitive deficits (Livingston et al., 2017). Various factors have been identified, including cardiovascular risk factors (e.g., hypertension, diabetes, and obesity), psychosocial factors (e.g., depression and social isolation), and health behaviors (e.g., physical inactivity, smoking, and hearing loss) (Livingston et al., 2017). Studies that have examined the association between such factors and WM structure are backed by theories of brain reserve (Bartrés-Faz and Arenaza-Urquijo, 2011, Stern, 2012)*,* which suggest that higher levels of, for example, WM (such as volume or microstructure) correspond to a better tolerance of age- and disease-related damage. Whereas the relationship between modifiable factors and age-related changes in the brain's gray matter has been the subject of reviews (Arenaza-Urquijo et al., 2015, Fotuhi et al., 2012), the association between lifestyle factors and WM has received much less attention.

To inform discussions and recommendations on how best to promote healthy brain aging, it is important to consider the extent to which modifiable factors can prevent, slow, or even reverse age-related WM decline. In this review, we focus on evidence from diffusion tensor imaging (DTI) studies that have examined modifiable factors proposed to alter the brain's WM microstructure during aging. We begin by highlighting common DTI measures of WM microstructure and their age-related patterns of change. Then, for each modifiable factor, we first summarize findings of studies that have examined whether there is a relationship between the factor and WM microstructure, before assessing the evidence of whether modifying the factor also modifies WM microstructure. Finally, we discuss how this field can contribute to public health policy going forward.

## 2 Search strategy and selection criteria

This review summarizes the evidence regarding the relationship between WM microstructure and a range of modifiable risk factors in healthy aging populations (mean age above 60 year old). We included both established modifiable risk factors for dementia that have been highlighted by major reviews in this field (hearing loss, hypertension, obesity, diabetes, smoking, depression, physical (in) activity, and social isolation (Barnes and Yaffe, 2011, Livingston et al., 2017)) and also emerging modifiable factors that have been linked to cognitive decline and dementia (sleep disturbances [Yaffe et al., 2016], cognitive training [Livingston et al., 2017], diet [Livingston et al., 2017], and meditation [Russell-Williams et al., 2018]). We identified DTI studies of these modifiable factors by searches of PubMed database between January 1, 2012, and March 20, 2019, and references from relevant articles. A combination of the following search terms was used: “white matter integrity or microstructure”, “diffusion tensor imaging”, “ageing”, “older adults”, “modifying factors or risk factors”, “hearing loss or presbycusis”, “vascular factors”, “hypertension”, “overweight or obesity”, “diabetes”, “social isolation or loneliness or social activity”, “smoking”, “sleep”, “sub-clinical or sub-threshold depression”, “physical activity”, “diet or nutrition”, “cognitive training”, “meditation”. There were no language restrictions. We prioritized studies with a sample size of above 100 for cross-sectional studies, or above 50 for longitudinal studies or randomised controlled trials, for each modifiable factor. If there was a small number of well-sampled studies, we widened our review to consider evidence from studies with smaller sample sizes or younger mean age.

## 3 Measuring WM microstructure

While, traditionally, MR studies in the field of aging and dementia have examined WM macrostructure, such as WM hyperintensities, DTI has expanded the field of research by providing a means for the noninvasive and in vivo examination of WM microstructure (Le Bihan and Johansen-Berg, 2012) (see Supplementary Material for alternative methods of WM imaging). DTI can reveal subtle impairments in WM microstructural before they can be detected using conventional MR methods (Prins and Scheltens, 2015), thus providing complementary information to traditional MR methods that may help earlier (or differential [Suri et al., 2014]) diagnosis of disease and advance our understanding of WM degeneration.

**Fig. 2:**
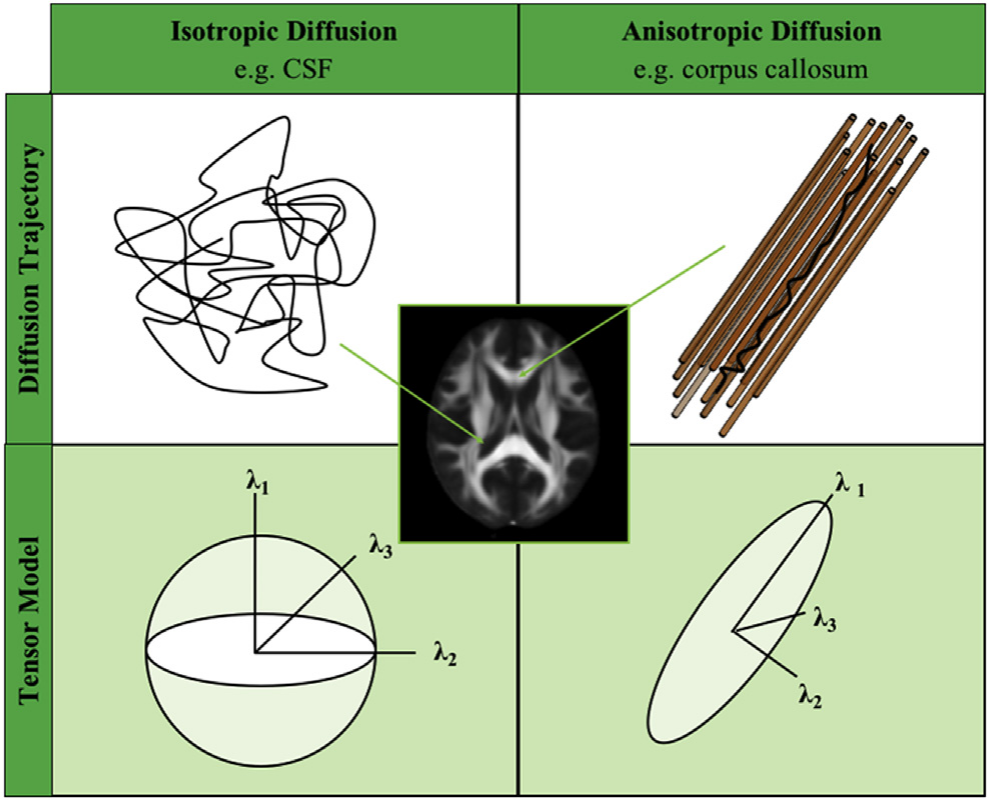
Isotropic and anisotropic diffusion and the tensor model. The diffusion trajectory (*top row*) of water is different in the presence or absence of barriers. The tensor model describes water diffusion at each voxel *(bottom row).* In the model, water diffusion is characterized by its 3 principal eigenvectors and their associated eigenvalues (λ1, λ2, λ3). The DTI parameters—FA, MD, RD, and AxD—can be computed from the eigenvalues of the tensor. Fractional anisotropy (FA) reflects the shape of the tensor and has higher values with more ellipsoid shape tensors (i.e., with anisotropic diffusion—*bottom right*)*.* Mean diffusivity (MD) is the magnitude of diffusion and can be computed by averaging the eigenvalues of the tensor [(λ1 + λ2 + λ3)/3]. Radial diffusivity (RD) reflects the diffusion perpendicular to the long axis of the tensor and can be computed by averaging eigenvalues λ2 and λ3 [(λ2 + λ3)/2]. Axial diffusivity (AxD) reflects the diffusion along the long axis of the tensor and is equal to λ1. (For interpretation of the references to color in this figure legend, the reader is referred to the Web version of this article.)

DTI is sensitive to the diffusion of water molecules, which is dependent on the presence or absence of barriers in neural tissue (Fig. 2). If no barriers are present, such as in the cerebrospinal fluid, water diffuses uniformly in all directions (i.e., isotropic diffusion). By contrast, if water movement is restricted in any direction, diffusion tends to follow the long axis of those barriers (i.e., anisotropic diffusion). Within WM, microstructural barriers such as axonal cell membranes and myelin sheaths restrict perpendicular water diffusion, causing the primary direction of diffusion to run along the fiber bundle.

By fitting a diffusion tensor model to each voxel, several useful parameters can be computed that provide information about WM microstructure. The most common metrics are fractional anisotropy (FA) and mean diffusivity (MD). FA represents the fraction of the tensor that can be ascribed to anisotropic diffusion and ranges from zero to one, with higher FA values reflecting increased diffusion directionality. MD reflects the magnitude (i.e., rate) of water diffusion, with higher values denoting increased diffusion. Additional information can be gained from the diffusivities parallel (axial diffusivity, AxD) or perpendicular (radial diffusivity, RD) to the long axis of the tensor.

These metrics may be compared across the whole brain using voxel-based analysis (VBA) or tract-based spatial statistics, or locally in a priori–defined regions using a region of interest (ROI) or tractography-based approach (O'Donnell and Pasternak, 2015). VBA involves spatially normalizing participants' scans to a standard space and performing voxelwise between-group statistics but is limited by the accuracy of spatial normalization and the amount of spatial smoothing of data (Abe et al., 2010, Jones et al., 2005). Tract-based spatial statistics determines an average FA tract skeleton onto which all participant's FA data are projected, before applying voxelwise statistics (Smith et al., 2006). This approach requires no smoothing and minimizes registration problems, which makes it more robust and sensitive than VBA, yet it limits the analysis to the core of the WM (Smith et al., 2006). On a local level, ROI and tractography approaches have been used to examine a priori–defined regions. ROI analysis involves manual or automatic (atlas-based) delineation of an ROI and its placement over a predefined region to extract some statistic of interest (e.g., mean value). This method avoids registration errors, but cannot detect localized changes within structures and manual placement is susceptible to user bias. Tractography algorithms use local information on orientation to reconstruct WM tracts, by propagating from a specific source region or between a source and target region. Although this method allows subject-specific tracts to be traced and quantified, its tracking is not always accurate (i.e., false positive and negative connections) and manual delineation of seed regions could be prone to user bias (Jbabdi et al., 2015, Jbabdi and Johansen-Berg, 2011).

Although DTI is highly sensitive to changes in WM microstructure and provides an excellent marker for microstructural change, it lacks specificity toward WM tissue properties, such as myelination or axon diameter (Alexander et al., 2017, Jones et al., 2013, Novikov et al., 2018b). FA, for instance, is sensitive to a multitude of WM features, including myelination, fiber density, fiber organization, and axonal degeneration (Beaulieu, 2009). More distinct anatomical features have been related to AxD and RD, with animal studies suggesting that lower AxD is related to axonal injury and higher RD reflects demyelination (Song et al., 2003, Sun et al., 2006). It is now well-accepted, however, that DTI metrics can be affected by various factors, including fiber arrangement, axon density, and cell swelling (Jones et al., 2013, Tournier et al., 2011, Wheeler-Kingshott and Cercignani, 2009), which limit the biological interpretability of these metrics. In general, however, a decline of WM structure (e.g., loss of myelin) results in lower WM directionality, reflected by lower FA and higher MD values (Bennett and Madden, 2014). Alternative biophysical models have recently been developed to better assess WM tissue microstructure (see Supplementary Material) (Alexander et al., 2017, Novikov et al., 2018a).

## 4 White matter microstructure, aging, and dementia

It is now well established that WM integrity declines with age (Bennett and Madden, 2014). This process typically begins between the third and fifth decade of life (Kochunov et al., 2012, Lebel et al., 2012, Sexton et al., 2014), is thought to accelerate with age (Sexton et al., 2014), and is associated with a high degree of interindividual variability (Bender and Raz, 2015). Across many cross-sectional DTI studies of aging, the general trend of findings is a global decrease in FA and increase in MD and RD values as a function of increasing age, whereas changes in AxD have been less consistent (Bennett and Madden, 2014, Cox et al., 2016, de Groot et al., 2015). Support for these results has recently been provided by longitudinal studies (Bender et al., 2016, Bender and Raz, 2015, de Groot et al., 2016, Sexton et al., 2014) that can account for individual differences at the baseline and thus better characterize age-related changes in WM microstructure. In general, the pattern of WM changes suggested by these studies is consistent with postmortem histological studies that have shown degeneration and deformation of axons and myelin with aging (Peters, 2002).

Age-related decline of WM integrity has also been examined on a regional or tract-specific level. Although findings vary by the type of study (i.e., cross-sectional vs. longitudinal) (Bender and Raz, 2015), the DTI analysis technique used (e.g., voxelwise or tract-based), and the age range of the sample included, some regions have shown to be consistently affected with aging. Specifically, it has been proposed that age-related degeneration is particularly prevalent within the frontal lobe (Bennett and Madden, 2014), follows posterior-to-anterior (Sullivan and Pfefferbaum, 2006) and inferior-to-superior (Sexton et al., 2014) gradients of lesser-to-greater vulnerability, and predominantly affects thalamic radiations and/or association fasciculi (Bender et al., 2016, Benitez et al., 2018, Brickman et al., 2012, Cox et al., 2016). Such findings have led researchers to postulate the retrogenesis hypothesis, which states that late-myelinating tracts, such as the association fibers, are the first to degenerate with aging (Bartzokis, 2004, Brickman et al., 2012). Crucially, integrity of these affected tracts has been associated with various cognitive functions (Madden et al., 2012) and loss of their integrity is thought to contribute to age-related cognitive decline.

Loss of WM integrity is also a feature of the common dementia subtypes, such as AD, Lewy-body dementia, vascular dementia, and frontotemporal dementia (Clerx et al., 2012, Sexton et al., 2011, Suri et al., 2014, Yin et al., 2015, Yu et al., 2017). A decline in integrity of major WM tracts, including the association fasciculi, has been reported in all these subtypes (Suri et al., 2014), may precede GM atrophy (Sachdev et al., 2013), and could help explain the well-described cognitive deficits. Interestingly, several studies demonstrate that the degree of WM microstructural abnormalities may be related to dementia severity (Amlien and Fjell, 2014, Suri et al., 2014), which could further improve monitoring of disease progression.

## 5 Modifiable factors and WM microstructure

Previous studies have identified a set of common mid- to late-life modifiable factors that are linked to an increased risk for dementia (Barnes and Yaffe, 2011, Livingston et al., 2017). Established modifiable factors include hearing loss, hypertension, obesity, diabetes, smoking, depression, physical (in) activity, and social isolation. Here we discuss evidence for a relationship between these factors and WM integrity during aging. We also review modifiable factors that we consider to be upcoming and that are linked to cognitive decline and dementia, such as sleep disturbances (Yaffe et al., 2016), cognitive training (Livingston et al., 2017), diet (Livingston et al., 2017), and meditation (Russell-Williams et al., 2018).

### 5.1 Hearing loss

Hearing loss is common among older adults and is associated with cognitive impairment and dementia (Livingston et al., 2017). In cross-sectional studies involving young adults, hearing impairments are related with loss of WM integrity, but studies in older adults are still sparse (Tarabichi et al., 2017). In a small study (n = 29), lower FA and higher MD, RD, and AxD was found in parts of the auditory pathway of older adults with age-related hearing loss (Ma et al., 2016). No such relationships were found, however, in a similarly small study (n = 44) comparing older adults with mild or severe age-related hearing loss and young controls (Profant et al., 2014). In a recent population-based study of middle-aged to older adults (Rigters et al., 2018), worse hearing was correlated with lower FA and higher MD, independent of age, WM lesions, or cardiovascular factors (e.g., blood pressure). Regionally, lower FA was observed in the superior longitudinal fasciculus, as well as lower FA and higher MD in the uncinate fasciculus. Although the evidence is limited, these results, and findings in young adults, suggest that hearing impairments may be related to compromised WM integrity. However, whether lower WM integrity is the cause or the effect of hearing loss cannot be established; longitudinal studies are needed to confirm these findings. It would also be of interest to explore whether WM loss is reversible with treatment such as hearing aids, despite some evidence suggesting that such devices may not improve cognitive performance (Murphy, 2019).

### 5.2 Hypertension

Hypertension, or high blood pressure, in middle-aged and older adults is related to DTI metrics that reflect compromised WM integrity, particularly lower FA and higher MD values (Allen et al., 2016, Bender and Raz, 2015, de Groot et al., 2015, Gons et al., 2012, Hannawi et al., 2018, Maillard et al., 2012, Mcevoy et al., 2015, Power et al., 2017, Salat et al., 2012, Suzuki et al., 2017) (but see the study by Bender et al., 2016, de Groot et al., 2016, Sabisz et al., 2019). Interestingly, the associations between blood pressure and FA and MD are already detectable in preclinical stages (Maillard et al., 2012, Salat et al., 2012) (but see the study by Suzuki et al., 2017), and remain significant after controlling for age and WM lesions (Power et al., 2017, Salat et al., 2012). Higher arterial stiffness has been identified as a cause of higher blood pressure, which increases with aging, and has shown a negative association with WM integrity (Maillard et al., 2016). A recent study reported a significant hypertension by age interaction, suggesting accelerated WM loss (reflected by higher MD, AxD, RD) in middle-aged hypertensive adults, but only in the left hemisphere (Sabisz et al., 2019). Findings from a small number of longitudinal studies that have examined the relationship between WM integrity and hypertension over a period of 2 to 10 years have, however, been inconsistent. In one study, lower FA was found in older participants with higher mean and more variable blood pressure trajectories compared to those with more stable trajectories (adjusted for age, race, and cardiometabolic markers) (Rosano et al., 2015). However, other studies reported no relationship between diagnosed hypertension and (change in) FA or diffusivity measures (Bender et al., 2016, de Groot et al., 2016), or even higher FA, albeit in a cohort combining young and older adults (Bender and Raz, 2015). Associations between hypertension and WM integrity follow spatial patterns that are similar to those reported for aging. For example, lower FA and higher MD values have been reported in anterior parts of the corpus callosum (de Groot et al., 2015, Hannawi et al., 2018, Maillard et al., 2012, Salat et al., 2012) (but see the study by Power et al., 2017), anterior thalamic radiation (Carnevale et al., 2018, Mcevoy et al., 2015), in various association tracts (Allen et al., 2016, Carnevale et al., 2018, de Groot et al., 2015, Hannawi et al., 2018, Maillard et al., 2012, Mcevoy et al., 2015, Rosano et al., 2015, Suzuki et al., 2017), particularly the uncinate and superior longitudinal fasciculi, as well as in more central and posterior regions (Power et al., 2017, Salat et al., 2012). Only a handful of studies have examined the relation of hypertension with RD and AxD, generally showing positive associations in regions similar to those reported for FA (Bender and Raz, 2015, Carnevale et al., 2018, Mcevoy et al., 2015, Salat et al., 2012).

Several studies have suggested that treatment of hypertension may slow down or reverse WM abnormalities. Cross-sectional studies have found reduced associations between measures of blood pressure and FA in treated compared with untreated participants (Maillard et al., 2016, Salat et al., 2012), possibly reflecting a therapeutic effect of treatment on WM damage (Table 1). However, higher WM integrity in untreated compared with treated participants has also been reported (Gons et al., 2012, Suzuki et al., 2017), possibly because medicated hypertensives had more sustained and severe hypertension that resulted in irreversible WM damage before being treated (Suzuki et al., 2017). In addition, higher WM integrity has been found for participants receiving adequate versus inadequate treatment (Gons et al., 2012), although other studies demonstrated no such differences (Mcevoy et al., 2015). A longitudinal study reported that longer duration of hypertension treatment was related to increased FA in the corpus callosum over time (Bender and Raz, 2015), suggesting that early intervention may prevent WM deterioration. In women only, however, longer treatment for hypertension was related to lower baseline FA of the forceps minor and increased FA decline of the superior longitudinal fasciculus (Bender and Raz, 2015). Given that a recent study has shown that blood pressure control reduces risk of mild cognitive impairment (Williamson et al., 2019), it will be of interest if intervention studies include imaging outcomes, such as diffusion MRI, as part of their assessments. Indeed, findings from a randomized open-label trial may elucidate the effects of antihypertensive therapy on WM integrity in older adults (White et al., 2013).

**Table 1:** Overview of the evidence for modification of risk factors on WM integrity

Study	Sample	Design	DTI	Main findings
Hearing loss		No evidence, need for longitudinal studies that examine the effects of hearing aids		
Hypertension		Preliminary evidence for treatment of hypertension, mainly from cross-sectional studies		
Gons et al. (2012)	499 older adults with CSVD; aged 50–85 y 271 on medication	Cross-sectional	ROI (1.5 T)	Lower FA and higher MD in CC individuals without adequate treatment compared with those with adequate treatment. Note, higher WM integrity in untreated compared with treated individuals.
Salat et al. (2012)	128 older adults aged 43–87 y 61 on medication	Cross-sectional	ROI on TBSS skeleton (1.5 T)	Significant blood pressure by medication status interactions were found in various regions, showing reduced associations with FA in individuals using antihypertensive medication compared to those that were not using medication.
Maillard et al. (2016)	1903 adults aged 24–76 y 328 on medication	Cross-sectional	Voxelwise (1.5)	Significant interaction of aortic stiffness with medication, suggesting a reduced association of aortic stiffness with FA in CR individuals receiving antihypertensive treatment (controlled for age and sex).
Suzuki et al. (2017)	4659 adults; mean age 62.3 y 1047 on medication	Cross-sectional	Tractography (3T)	Lower global FA and higher MD in medicated compared with unmedicated individuals. Furthermore, lower FA was found in the SLF, anterior TR (right) and posterior TR (left), Fmin, as well as higher MD in the SLF, ILF, anterior TR (left) and acoustic radiation (left) (groups were matched on several covariates).
Bender and Raz (2015)	96 adults aged 19–78 y	Longitudinal (2 y follow-up)	ROI on TBSS skeleton (4T)	More years of hypertension treatment were related to greater increases in FA in the CC (body). Furthermore, longer duration of treatment was significantly related to lower baseline forceps minor FA and increased SLF FA decline for women, but not men (corrected for age, interval between scans).
Diabetes		No evidence, need for longitudinal studies that examine the effects of diabetes medication		
Obesity		Promising evidence for treatment of obesity, but not in older adults		
Krafft et al. (2014) Schaeffer et al. (2014)	18 overweight children aged 8–11 y old	8-mo after school intervention of either (1) aerobic exercise, or (2) sedentary attention control	Tractography (3T)	Krafft et al. (2014): significant time by group attendance interaction, showing higher FA and lower RD in SLF with higher attendance in the exercise group only (corrected for age and sex). Schaeffer et al. (2014): Higher FA and lower RD in, respectively, bilateral and left UF after exercise (controlled for race and sex).
Zhang et al. (2016)	33 adults, of which 15 morbidly obese; mean age 25.8 y (obese) and 27 y (controls)	MRI scan before and 1-mo after bariatric surgery in morbidly obese	TBSS (3T)	Before surgery, the morbidly obese had lower FA and higher MD compared with normal weight controls. From pre- to post surgery, the morbidly obese individuals had higher FA in CR, CC (body, genu), FNX, ST, ILF, IFOF, and lower MD in left CR, left EC, left IC, left SLF and left sagittal stratum (ILF, IFOF) (controlled for age, sex, anxiety and depression).
Smoking		Promising evidence, but cross-sectional designs		
Gons et al. (2011)	503 older adults with CSVD; aged 50–85 y	Cross-sectional	Whole brain (1.5 T)	A significant association between the length of smoking cessation and lower MD and higher FA values (controlled for age, sex, alcohol intake, education, and cardiovascular risk factors).
de Groot et al. (2015)	4532 aduls; aged 45.7–100 y	Cross-sectional	Tractography (1.5 T)	Current smokers had significantly lower FA in Fmin and CST compared with former smokers, but not individuals who never smoked. Current smokers had higher MD in Fmin, CST, medial lemniscus, superior TR compared with never or former smokers; no significant differences between former or never smokers (controlled for age, sex, ICV and tract-specific volume and WM lesion volume).
Depressive symptoms		Promising evidence from research into major depression		
Wang et al. (2013)	43 adults, of which 21 MDD; mean age 29.6 y (MDD)	MDD patients received 4-wk of psychotherapy	Voxelwise (3T)	After treatment, patients had higher FA in the left superior frontal cortex and lower FA in the right angular cortex WM (corrected for age and sex).
Lyden et al. (2014)	28 adults, of which 20 MDD; mean age 41.15 y (MDD)	MRI scans before, after the second and within 1 wk of completion of electroconvulsive therapy (ECT) series in MDD patients	TBSS (3T)	MDD patients had significantly higher FA and lower MD and RD in anterior CIN, Fmin, left SLF between baseline the third time point of ECT (transition to maintenance therapy), as well as lower MD and RD in anterior TR (corrected for age and sex).
Social isolation		Preliminary evidence from research into socially engaging activities		
Molesworth et al. (2015)	155 adults; mean age 40.7 y	Cross-sectional	Tractography (3T)	Higher global FA was related to diversity of a person's social network (controlling for age, sex, education, and central adiposity).
Köhncke et al. (2016)	70 older adults time point 1, 37 older adults time point 2; aged >81 y	Longitudinal (3-y follow-up)	TBSS (1.5 T)	Higher FA and lower MD in the CST is related to higher engagement in social activities (change–change association); no relationships between WM integrity and social activities were found at baseline (corrected for age, sex, and education).
Sleep		Preliminary evidence from research into sleep apnea and sleep medication		
Castronovo et al. (2014)	28 adults, of which 13 obstructive sleep apnea; aged 30–55 y	MRI before, after 3 and 12 mo of CPAP treatment	TBSS (3T)	Patients had lower FA and MD at baseline compared with controls, in various regions, including the SLF, UF, deep frontal WM. After 3 mo of CPAP, lower FA and MD were detected in similar regions, such as SLF and UF, but to a lesser extent. After 12-mo of CPAP, lower FA and MD were only still visible in the SLF, but to a lesser extent than at 3 mo or baseline.
Gadie et al. (2017)	641 adults; aged 18–98 y	Cross-sectional	ROI (3T)	Sleep medication was associated with higher FA in CST, as well as higher FA in SLF of older adult (age by sleep interaction).

Key: AxD, axial diffusivity; AR, acoustic radiation; CC, corpus callosum; CIN, cingulum; CPAP, continuous positive airway pressure; CR, corona radiata; CST, corticospinal tract; CSVD, cerebral small-vessel disease; CT, cognitive training; DTI, diffusion tensor imaging; EC, external capsule; ECT, electroconvulsive therapy; FA, fractional anisotropy; Fmin, forceps minor; FNX, fornix; IC, internal capsule; ICV, intracranial volume; ILF, inferior longitudinal fasciculus; IFOF, inferior fronto-occipital fasciculus; MD, mean diffusivity; MDD, major depressive disorder; RD, radial diffusivity; ROI, region of interest; SLF, superior longitudinal fasciculus; ST, stria terminalis; TBSS, tract-based spatial statistics; TR, thalamic radiation; UF, uncinate fasciculus; WMH, white matter hyperintensities.

### 5.3 Obesity

White matter (WM) integrity has shown to be particularly vulnerable to the effects of obesity (Kullmann et al., 2015). Across many cross-sectional studies, obesity and higher BMI have been associated with lower whole-brain and tracts-specific FA values in older (Bettcher et al., 2013, Bolzenius et al., 2015, Ryan and Walther, 2014), but also middle-aged adults (Allen et al., 2016, Kullmann et al., 2015, Papageorgiou et al., 2017, Repple et al., 2018, Spieker et al., 2015, Zhang et al., 2018) (but see the study by Birdsill et al., 2017, Hannawi et al., 2018). Interestingly, a negative association has also been reported between higher abdominal fat mass, but not BMI, and FA (Ryan et al., 2017), and lower WM integrity (i.e., lower FA) is already present in obese nondiabetic adolescents with metabolic syndrome (Yau et al., 2012). Diffusivity measures have shown a less consistent pattern, with generally lower AxD and mixed results for RD and MD (Allen et al., 2016, Kullmann et al., 2016, Ryan and Walther, 2014, van Bloemendaal et al., 2016). Regionally, the corpus callosum, particularly the genu, has shown WM degeneration with higher BMI, reflected in lower FA, higher RD, and lower AxD values (Allen et al., 2016, Bettcher et al., 2013, Papageorgiou et al., 2017, Ryan and Walther, 2014, Spieker et al., 2015, Stanek et al., 2011, Zhang et al., 2018). These effects remained after controlling for age and vascular and inflammatory markers (Bettcher et al., 2013, Repple et al., 2018, Zhang et al., 2018). In addition, a significant BMI by age interaction was found within the corpus callosum in one study (Stanek et al., 2011), but not other studies (Bolzenius et al., 2015, Zhang et al., 2018), indicating that well-controlled longitudinal studies are now needed to examine whether obesity exacerbates age-related WM decline. Other regions that show WM degeneration with higher BMI are the cingulum (Bettcher et al., 2013, Papageorgiou et al., 2017), the fornix (Bettcher et al., 2013, Stanek et al., 2011), and corona radiata (Kullmann et al., 2015, Ryan and Walther, 2014, Zhang et al., 2018), as well as several association fibers, including the uncinate, superior longitudinal, and inferior fronto-occipital fasciculi (Bolzenius et al., 2015, Karlsson et al., 2013, Papageorgiou et al., 2017, Repple et al., 2018).

To our knowledge, no clinical trial has been conducted to determine the effect of obesity treatment on WM integrity in older adults. In overweight children, intervention studies have shown a beneficial effect of exercise and attendance at an exercise program on WM integrity of the uncinate fasciculus (Schaeffer et al., 2014) and superior longitudinal fasciculus (Krafft et al., 2014), respectively. A recent study has shown recovery of WM integrity abnormalities in morbidly obese individuals 1 month after bariatric surgery (Zhang et al., 2016). These preliminary studies involving small groups of young people suggest that obesity-related WM degeneration is reversible and highlight the need for intervention studies in older adults.

### 5.4 Diabetes mellitus

A decline in WM integrity has consistently been reported in patients with diabetes mellitus (Biessels and Reijmer, 2014). This negative relationship is true for both diabetes type I (Biessels and Reijmer, 2014), which commonly has a childhood onset, and for type II (Falvey et al., 2013, Hoogenboom et al., 2014, Hsu et al., 2012, Reijmer et al., 2013, Tan et al., 2016, van Bloemendaal et al., 2016, Xiong et al., 2016, Zhang et al., 2014), which is more common among older adults, and is therefore the focus of this section. Most studies, all cross-sectional in design, have shown a global and regional decrease in FA and increase in MD values in middle- to old-aged patients with type II diabetes compared with age-matched controls (Falvey et al., 2013, Hoogenboom et al., 2014, Hsu et al., 2012, Reijmer et al., 2013, Tan et al., 2016, Xiong et al., 2016), as well as higher RD and AxD values, albeit less consistently (van Bloemendaal et al., 2016, Zhang et al., 2014). The associations remain significant after controlling for differences in hypertension, BMI, and WM hyperintensities (Falvey et al., 2013, Reijmer et al., 2013). Longer disease duration was found to be related to worse WM integrity, indicated by higher diffusivity values (Hsu et al., 2012). Regionally, changes in WM with type II diabetes vary between studies, likely because of differences in sample characteristics (e.g., age, size) and analysis techniques used. For example, in one study, using tractography in older adult diabetics and nondiabetics, lower FA, and higher MD, RD, and AxD in the uncinate fasciculus have been reported (Reijmer et al., 2013), as well as diffusivity differences in inferior and superior longitudinal fasciculi. Diabetes-related degeneration of these tracts has been confirmed by other studies (Hoogenboom et al., 2014, Tan et al., 2016, Zhang et al., 2014). Abnormalities have also been found in frontal WM (Falvey et al., 2013, Hsu et al., 2012), the thalamic radiation (Tan et al., 2016, Zhang et al., 2014), and the internal capsule and corona radiata (Xiong et al., 2016, Zhang et al., 2014)—tracts known to degenerate with aging. Longitudinal studies are needed to determine the effects of diabetes medication on WM integrity.

### 5.5 Smoking

The association between tobacco smoking and WM integrity is complex. Cross-sectional studies examining WM integrity in middle-aged (Lin et al., 2013, Savjani et al., 2014, Umene-Nakano et al., 2014, Viswanath et al., 2015) and older (de Groot et al., 2015, Gons et al., 2011) smokers have shown lower FA and higher MD values compared with nonsmokers or former smokers, even after correction for age, blood pressure, BMI, diabetes, and alcohol consumption (Gons et al., 2011). In addition, longer duration of smoking has been related to worse WM integrity (Baeza-Loya et al., 2016, Lin et al., 2013, Umene-Nakano et al., 2014, Viswanath et al., 2015). Tract-specific changes have most frequently been observed in the corpus callosum and internal capsule, showing lower FA in smoking adults (de Groot et al., 2015, Lin et al., 2013, Savjani et al., 2014, Umene-Nakano et al., 2014, Viswanath et al., 2015). MD, RD, and AxD changes have been examined less often, but seem to support WM degeneration in those tracts (i.e., higher MD and RD, lower AxD). While such results are consistent with other vascular risk factors, they contrast to studies in adolescents and young adults, which have consistently shown increased FA values in smokers compared with nonsmokers (Gogliettino et al., 2016). In addition, acute administration of nicotine (Kochunov et al., 2013) and light-smoking or low nicotine dependence in middle-aged adults (Hudkins et al., 2012) have been related to higher FA values. One hypothesis for this discrepancy is that there are deleterious effects of chronic, heavy-smoking on WM integrity with aging (Kochunov et al., 2013). However, this hypothesis is yet to be examined by longitudinal studies.

Some evidence suggests that quitting smoking may reverse impaired WM integrity. A cross-sectional study has shown that FA and MD values of participants who quit smoking (>20 years ago) were comparable with those who never smoked (Gons et al., 2011). In another study, lower FA was found in the forceps minor and corticospinal tract for current compared with former smokers (de Groot et al., 2015).

### 5.6 Depressive symptoms

While many studies have reported WM degeneration in late-life major depression, particularly in the frontal lobe, corpus callosum, uncinate fasciculus, and cingulum (Wen et al., 2014), it has recently become clear that WM decline is also present in older adults with subthreshold depression (Allan et al., 2016, Hayakawa et al., 2013, Hayakawa et al., 2014, McIntosh et al., 2013, Tudorascu et al., 2014). The available evidence consistently indicates lower global FA in large samples (N > 300) of old adults with subthreshold depression (Allan et al., 2016, Hayakawa et al., 2014, McIntosh et al., 2013, Tudorascu et al., 2014), and some studies have found support for higher MD, RD, and AxD (Allan et al., 2016, Hayakawa et al., 2013). Subthreshold depressive symptoms have primarily been associated with degeneration of frontal WM tracts, such as the anterior cingulum, corpus callosum, and uncinate fasciculus (Allan et al., 2016, Hayakawa et al., 2014, McIntosh et al., 2013, Tudorascu et al., 2014), but temporal and occipital tracts have been implicated too (Allan et al., 2016, Tudorascu et al., 2014).

Although no subthreshold depression intervention studies have been published to date, treatment of major depression demonstrates reversibility of impaired WM integrity. Studies using electroconvulsive therapy (Lyden et al., 2014) and psychotherapy (Wang et al., 2013) have shown higher FA after treatment; the former showing increased WM integrity in regions typically affected in major depression. Hence, future intervention studies in subclinically depressed patients are desired.

### 5.7 Physical activity

Physical activity and fitness are widely regarded as important factors for cognitive and brain health, and current evidence suggests that these factors preserve WM integrity in older adults. A recent systematic review indicated higher FA in physical active or fit individuals, with less, but consistent, evidence for either lower or unchanged diffusivity values (MD, RD, AxD) (Sexton et al., 2016). Regionally, higher levels of physical activity or fitness have been related to higher FA in many tracts, but most consistently in the corpus callosum (Burzynska et al., 2015, Hayes et al., 2015, Oberlin et al., 2016, Tian et al., 2015, Verkooijen et al., 2017), corona radiata (Oberlin et al., 2016, Smith et al., 2016, Verkooijen et al., 2017), fornix (Oberlin et al., 2016), internal capsule (Burzynska et al., 2015, Oberlin et al., 2016, Smith et al., 2016), cingulum (Oberlin et al., 2016, Tian et al., 2015), and several association tracts, among them the superior longitudinal (Oberlin et al., 2016, Tian et al., 2015), inferior fronto-occipital (Tian et al., 2015), and uncinate fasciculi (Burzynska et al., 2015). Diffusivity findings are less clear, with some studies showing that RD reductions accompany FA increases (Smith et al., 2016) and others reporting lower MD or RD in areas unrelated to FA changes (Gons et al., 2013). Although the FA findings are promising, studies suffer from small sample sizes and methodological differences (e.g., in physical activity measures), and the cross-sectional nature of the studies limit causal inferences.

Recent longitudinal and intervention studies (Table 2) also provide some support for the beneficial effects of PA on WM integrity. A longitudinal study has demonstrated that older adults who maintained physical activity over a 10-year period show smaller increases in AxD (Best et al., 2017). Findings from an intervention study showed that, despite an absence of differences in DTI measures between the exercise and control groups after intervention, a greater percentage increase in fitness was associated with higher WM integrity (i.e., FA) in prefrontal, parietal, and temporal regions in the exercise group (Voss et al., 2013). Another trial demonstrated higher FA of the fornix after a 6-month dance intervention, whereas lower FA was observed for all other groups (including walking) (Burzynska et al., 2017). By contrast, a 10-week exercise intervention in a small sample (N = 14) of older adults at risk of dementia did not show changes in WM integrity, but this was likely limited in power (Fissler et al., 2017).

**Table 2:** Intervention studies of the effect of physical activity, diet, cognitive training and meditation on WM microstructure

	Sample	Intervention	DTI	Main findings
Physical activity				
Voss et al. (2013)	70 older adults; aged 55–80 y	One year of either (1) walking or (2) flexibility, toning, and balance training	TBSS and ROI (3T)	No significant differences between the groups, but increased aerobic fitness was associated with significant increases in prefrontal, parietal, and temporal FA in the walking group only (controlled for age, sex, and intervention attendance).
Burzynska et al. (2017)	174 older adults; aged 60–79 y	6 mo of either (1) dance, (2) walking, (3) walking + nutrition, (4) active control group (stretching and toning)	ROI on TBSS skeleton (3T)	A significant time by group interaction was found for the fornix, with higher FA for the dance group, but lower FA for other groups. This effect was driven by RD and MD, which increased to a lesser extent in the dance group compared with the other groups.
Fissler et al. (2017)	39 older adults at risk of dementia; aged >55 y	10-wk intervention of either (1) physical exercise: aerobic, strength, coordination, balance, and flexibility elements, or (2) cognitive training, or (3) passive control	ROI (1.5 T)	No effect of physical exercise on FA compared with control, but fitness level was positively associated with fornix FA at the baseline.
Cognitive training				
Engvig et al. (2012)	41 older adults; aged 42–77 y	8-wk of either (1) memory training, or (2) control group: living as usual	TBSS (1.5 T)	A decrease in FA in the control group compared to the memory training group was reported in areas overlapping the left anterior TR, IFOF, UF, and SLF (controlled for baseline measures).
Strenziok et al. (2014)	42 older adults; mean age 69 y	6 wk of either: (1) Brain Fitness (BF): auditory perception, (2) Space Fortress (SF): visuomotor/working memory, or (3) Rise of Nations (RON): strategic reasoning	TBSS (3T)	An increase in AxD was found in the Brain Fitness group compared with the other groups from the baseline to follow-up. Increased AxD in BF compared with SF group was reported in: IFOF, ILF, CC, and posterior TR. AxD additionally increased in the SLF, IC, and AR in BF compared with the RON group.
Lampit et al. (2015)	12 older adults; aged >65 y	12 wk of either (1) multi-domain cognitive training, or (2) active control: viewing videos and answering questions	TBSS (3T)	No significant differences between the groups over time.
Chapman et al. (2015)	37 older adults; aged 56–71 y	12 wk of either: (1) cognitive training: gist reasoning, or (2) wait-list control	Tractography and ROI (3T)	Increased FA in the UF of the cognitive training group compared with the control group.
Cao et al. (2016)	48 older adults; aged 65–75 y	12 wk of either (1) multidomain cognitive training, (2) single-domain cognitive training: reasoning, (3) control group.	TBSS (3T)	The control group had higher RD and MD, and lower FA in the posterior CR compared with the multidomain cognitive training group. No differences in DTI measures were found from the baseline to follow-up between the multi- and single-domain cognitive training groups, nor the single-domain and control group (controlling for age, sex, education, and baseline DTI measures).
Antonenko et al. (2016)	25 older adults; age 56–78 y	3 d of object location training	ROI (3T)	Higher FA and significantly lower MD in the fornix, but not UF or cingulum, following 3-d of object location training.
de Lange et al. (2016)	104 older adults, mean age 73.5 y	10 wk of either (1) memory training, (2) active control: popular scientific lectures, (3) passive control	TBSS (3T)	Memory performance improved significantly in the memory training group only. Furthermore, negative relationships between memory improvement and MD, AxD, and RD were found in, for MD: anterior CC, the left anterior AR, and the right IFOF, for AxD: right IFOF, and for RD: anterior CC (controlled for age, sex and motion).
de Lange et al. (2017)	111 older adults; mean age 73 y	10 wk of either (1) memory training, (2) active control: popular scientific lectures, or (3) passive control	TBSS (3T)	The memory training group, relative to the control groups, showed an increase in FA and decrease in MD, RD, and AxD in areas overlapping the CC, the CST, the cingulum, the SLF and the anterior TR (controlled for age, sex, motion, baseline WM values and WM hypointensities).
Fissler et al. (2017)	39 older adults at risk of dementia; aged >55	10-wk intervention of either (1) physical exercise, (2) cognitive training: auditory processing and working memory, (3) passive control	ROI (1.5 T)	No effect of cognitive training on FA compared with the control group, but cognitive training skills at the baseline were associated with FA.
Diet				
Witte et al. (2014)	65 older adults; aged 50–75 y	26-wk intervention of (1) either fish oil (2.2 g/d LC-n3-FA) or (2) placebo	TBSS (3T)	Fish oil supplementation led to significant increases FA as well as decreases in MD and RD, in various areas: the anterior CC, the UF, the IFOF and SLF.
Meditation				
Tang et al. (2012)	*Study 1:* 45 young adults; mean age 20.58 y *Study 2:* 68 young adults; mean age 20.52 y	*Study 1:* 4 wk of either (1) integrative body-mind training (IBMT), or (2) relaxation training *Study 2:* 2 wk of either (1) IBMT, or (2) relaxation training	TBSS (3T)	*Study 1:* Increased FA and lower AxD and RD in the anterior and superior CR, anterior CC and SLF after IBMT but not relaxation training. *Study 2*: Decrease in AxD only in the CC, CR, SLF, posterior TR, and sagittal stratum following IBMT but not relaxation training.
Hölzel et al. (2016)	46 adults; mean age ∼32 y	8-wk of either (1) mindfulness-based stress reduction training, or (2) waitlist control	Tractography (3T)	Increased FA in right UF following meditation training, but no change in the control group and no significant group by time interaction.

Key: AD, axial diffusivity; AR, acoustic radiation; CC, corpus callosum; CR, corona radiata; CRT, corticospinal tract; CT, cognitive training; DTI, diffusion tensor imaging; FA, fractional anisotropy; IBMT, integrative body-mind training; IC, internal capsule; ILF, inferior longitudinal fasciculus; IFOF, inferior fronto-occipital fasciculus; MD, mean diffusivity; RD, radial diffusivity; ROI, region of interest; SLF, superior longitudinal fasciculus; TBSS, tract-based spatial statistics; TR, thalamic radiation; UF, uncinate fasciculus.

### 5.8 Social isolation

The relationship between social isolation and WM integrity has not received much attention in the literature and, to the best of our knowledge, has not been studied in older adults. A cross-sectional study in young adults (Tian et al., 2014) found a negative relationship between loneliness ratings and FA in 3 ROIs: external capsule, inferior fronto-occipital, and inferior longitudinal fasciculi. More recently, it was shown that the relationship between loneliness and WM integrity in young adults is modulated by the brain-derived neurotrophic factor Val66Met polymorphism. A negative relationship of loneliness with global FA and a positive relationship with global RD in Val/Met heterozygotes were found, but not Val/Val homozygotes (Meng et al., 2017). These relationships were detected across the brain, but particularly in the corpus callosum, corona radiata, and superior longitudinal fasciculus. These studies suggest that higher levels of loneliness are related with compromised WM integrity, albeit in young adults.

Encouragingly, in a cross-sectional study of middle-aged adults (Molesworth et al., 2015), a positive relationship was found between the diversity of a person's social network and FA, particularly of the anterior corpus callosum, after controlling for age, sex, education, and central adiposity. Furthermore, a longitudinal study demonstrated that increasing social activity engagement over a 3-year period was related to higher FA and lower MD in the corticospinal tract, whereas no relationship was found for MD (Köhncke et al., 2016). These studies suggest that a diverse network or higher levels of social activities may protect or enhance WM integrity, but intervention studies are needed to infer causality.

### 5.9 Sleep disturbances

With age, people have more difficulty falling asleep, sleep becomes more fragmented and total sleep duration decreases. Moreover, sleep disorders are more prevalent among older adults, particularly insomnia and obstructive sleep apnea (Yaffe et al., 2014). Both disorders have been associated with a decline in WM integrity (Chen et al., 2015, Li et al., 2016), with the internal capsule, superior longitudinal fasciculus, and corpus callosum being particularly affected in insomnia (Li et al., 2016). A growing body of work now also indicates that poor sleep is related to markers of lower WM integrity in nonclinical samples of older adults. Although findings vary by the sleep metric used (e.g., quality, duration), studies report that measures of poor sleep are associated with lower global FA and higher diffusivity values (Baillet et al., 2017, Gadie et al., 2017, Khalsa et al., 2017, Sexton et al., 2017). A recent longitudinal study, however, did not find evidence for a relationship between sleep complaints and changes in WM microstructure (Kocevska et al., 2019). Regionally, poor sleep has consistently been related with lower WM integrity in the internal capsule, corpus callosum, forceps minor, and superior longitudinal fasciculus (Baillet et al., 2017, Sexton et al., 2017)—tracts that are also affected in insomnia. The effects of poor sleep on WM integrity are similar during middle age, with shorter sleep duration being related to lower FA (Verkooijen et al., 2017, Yaffe et al., 2016) and higher MD in widespread areas (Yaffe et al., 2016). Interestingly, widespread decreases in FA have been detected after 1 day of sleep deprivation in young adults, indicating that poor sleep may impact on WM microstructure (Elvsåshagen et al., 2015). Conversely, it was shown that age-related WM degeneration in several tracts, including the corpus callosum, predicts a loss of characteristic oscillations of the sleeping brain (such as sleep spindles), suggesting that WM integrity may directly affect sleep itself (Mander et al., 2017). Such a bidirectional relationship between sleep and WM could lead to a downward spiral, with poor sleep leading to lower WM integrity, that in turn affects sleep.

Studies that examine the effects of sleep promoting factors (e.g., nonpharmacological sleep therapy, medication) on WM integrity are required to further examine directionality of this relationship. Preliminary evidence for a beneficial effect of sleep improvement on WM stems from a study showing that use of sleep medication was related to higher WM integrity (i.e., FA) of the corticospinal tract and superior longitudinal fasciculus (in older adults) (Gadie et al., 2017), as well as from a study demonstrating reversal of WM abnormalities after a 12-month continuous positive airway pressure treatment in individuals with sleep apnea (Castronovo et al., 2014).

### 5.10 Cognitive training

Cognitive training is effective for maintaining or improving cognitive performance in the domain trained in older adults and recent studies, predominantly interventional, suggest that single- and multi-domain cognitive training has beneficial effects on WM integrity. Several studies have examined the effect of memory training on WM microstructure in older adults (de Lange et al., 2016, de Lange et al., 2017, Engvig et al., 2012, Fissler et al., 2017), showing higher FA in the training compared with the control group after periods of 8 (Engvig et al., 2012) or 10 (de Lange et al., 2017, de Lange et al., 2016) weeks of training (but see the study by Fissler et al., 2017). FA changes were observed in the anterior corpus callosum, thalamic radiation, and several association fibers, including the uncinate, inferior fronto-occipital, and superior longitudinal fasciculi. Moreover, lower or smaller increases in MD, RD, and AxD were found in the training compared with the control group (de Lange et al., 2017, Engvig et al., 2012), which suggests a relative preservation of WM integrity with memory training.

Other studies have used a variety of multidomain problem-solving and logical reasoning training paradigms, lasting between 6 and 12 weeks, and showing either higher (Cao et al., 2016, Chapman et al., 2015) or no change (Lampit et al., 2015, Strenziok et al., 2014) in FA in the training group. One such study (Chapman et al., 2015) reported higher FA in the uncinate fasciculus in older adults after 12 weeks of gist reasoning training, but no changes in diffusivity measures. Another study (Cao et al., 2016) showed higher AxD after multidomain cognitive training in the intervention group, and lower FA and higher MD and RD in the control group, suggesting that training preserves WM integrity. Furthermore, it has recently been shown that 3 days of object-location training results in higher FA and significantly lower MD in the fornix, but not uncinate fasciculus, of older adults (Antonenko et al., 2016). Despite these promising results, however, training paradigms vary substantially between studies, sample sizes are small, and studies often lack replication, emphasizing the need for careful interpretation of results and further work in this area.

### 5.11 Diet

An increasing body of evidence suggests that dietary factors are associated with cognitive performance, and that diets, such as Mediterranean–Dietary Approach to Systolic Hypertension, have the potential to slow cognitive decline and reduce incidence of AD (Morris et al., 2015a, Morris et al., 2015b). A link between nutrition and brain structure has also been shown in older adults (Witte et al., 2014) and, recently, DTI studies have begun to shed light on the relationship between dietary factors, primarily polyunsaturated fatty acids (PUFAs) and WM microstructure. One such study reported that higher Mediterranean diet (i.e., plant foods, fish, monounsaturated fatty acids) adherence in older adults was associated with a pattern of preserved WM integrity, reflected by higher FA and lower diffusivity (MD, RD, AxD) in widespread WM areas, including the corpus callosum, anterior and posterior thalamic radiations and inferior fronto-occipital fasciculus (Pelletier et al., 2015). Another study showed that a nutrient pattern characterized by high intakes of Ω-3 and Ω-6 PUFAs and vitamin E, all of which are part of the Mediterranean diet, is related to higher global FA values (Gu et al., 2016).

A recent study that focused on PUFAs only has shown that higher levels of a mixture of Ω-3 and Ω-6 PUFAs is related to higher FA in the fornix of older adults (Zamroziewicz et al., 2018). In addition, a randomized controlled trial of a 26-week intervention of either fish oil (Ω-3 PUFA) or placebo supplementation in older adults has shown beneficial effects of Ω-3 PUFA supplementation over placebo on WM integrity, as indicated by higher FA and lower MD and RD in the anterior corpus callosum, and the uncinate, inferior fronto-occipital, and superior longitudinal fasciculi (Witte et al., 2014).

### 5.12 Meditation

Meditation has shown beneficial effects for WM integrity, although the available evidence is still sparse. In general, cross-sectional studies reported higher FA in meditators compared with nonmeditators (Kang et al., 2013, Laneri et al., 2016, Luders et al., 2012, Sharma et al., 2017) or in participants who score higher on a mindfulness scale (Boekel and Hsieh, 2018). Moreover, 2 studies have examined the relationship of FA with age in meditators and nonmeditators, showing that the general age-related decrease in WM integrity (reflected by FA) was less prominent in meditators than in nonmeditators (Laneri et al., 2016, Luders et al., 2011). A recent study supports these findings by showing that higher self-reported mindfulness may preserve FA in aging individuals (Boekel and Hsieh, 2018). The beneficial effects of meditation on WM integrity have been supported by intervention studies in young adults, showing higher FA after 4 weeks (Tang et al., 2012) and 8 weeks (Hölzel et al., 2016), but not 2 weeks (Tang et al., 2012), of mindfulness-based meditation training. Although current findings are promising, most studies have been conducted in small samples of young adults and lack insight into meditation-related diffusivity changes. On a regional level, meditators have shown higher FA in the corpus callosum, corona radiata, anterior thalamic radiation, and several association tracts (i.e., uncinate and superior-longitudinal fasciculi), tracts that are known to be affected by the aging process, although changes in other tracts have also been reported (Kang et al., 2013, Laneri et al., 2016, Luders et al., 2011, Luders et al., 2012, Sharma et al., 2017). Further longitudinal studies in older adults are needed to confirm any beneficial effect of meditation on WM integrity.

## 6 Discussion

### 6.1 Summary of findings

In this review, we examined various modifiable factors, all highly prevalent in older individuals, that have been linked with WM microstructure. Recognized risk factors, including vascular factors, such as hypertension, diabetes, obesity and smoking, depressive symptoms, social isolation, hearing loss, as well as sleep disturbances, seem to contribute to loss of WM integrity with age. Various other lifestyle factors, including physical activity, nutrition, cognitive training, and meditation, may preserve or protect WM integrity. Furthermore, there is preliminary evidence from cross-sectional studies of treated risk factors (Table 1) and interventional studies of protective factors (Table 2) which suggests that modification of factors may impact on WM integrity.

**Fig. 3:**
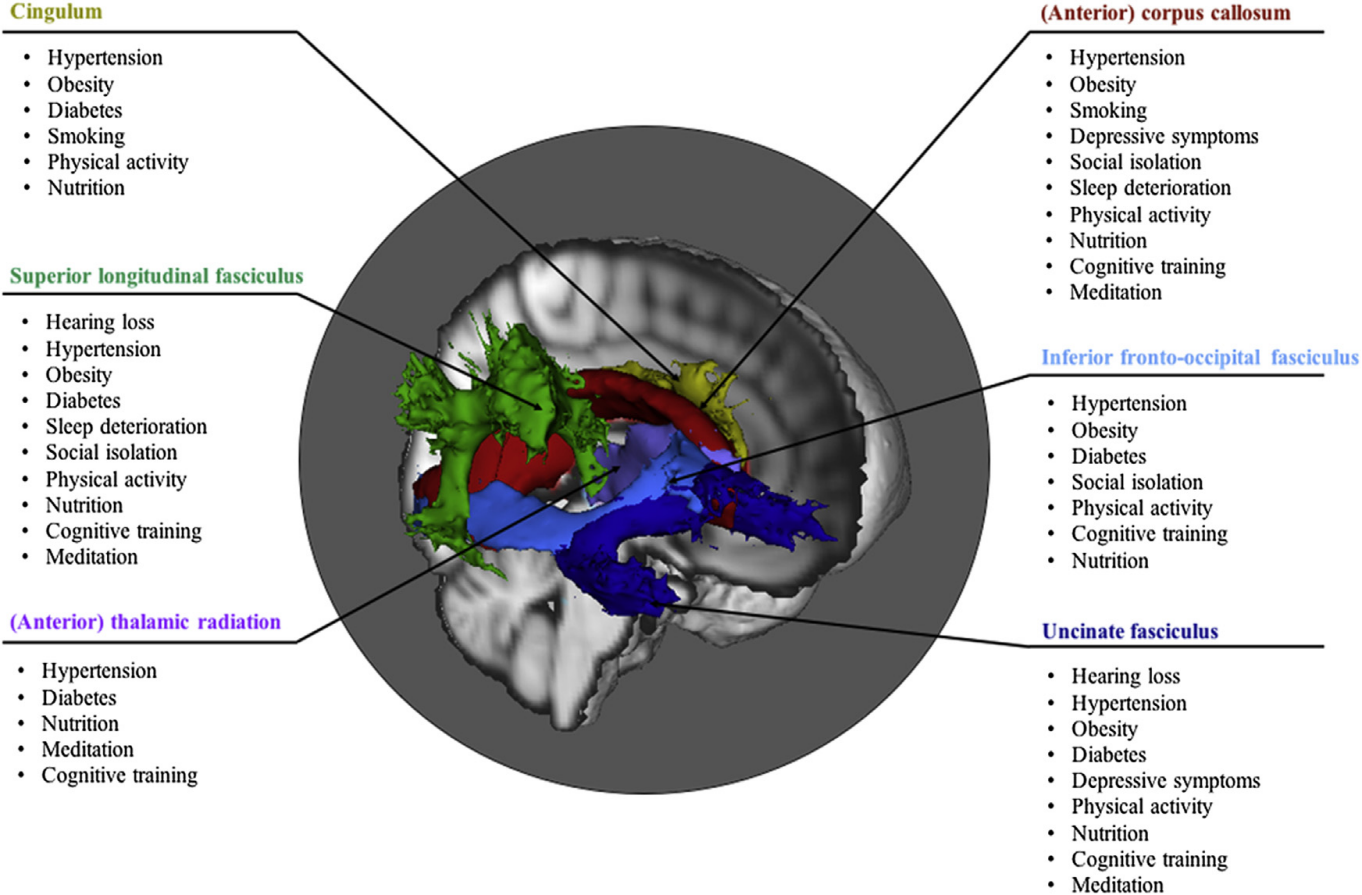
White matter tracts that are particularly sensitive to the identified modifiable factors. For each colored tract, factors are listed that have shown to be consistently associated with decline and/or protection of its integrity. These tracts have been implicated in various cognitive processes (Madden et al., 2012) and their integrity typically declines with age, suggesting a pathway whereby changing exposure to the factors could possibly prevent or slow age-related WM breakdown and cognitive decline. (For interpretation of the references to color in this figure legend, the reader is referred to the Web version of this article.)

Interestingly, a number of WM tracts seem to be particularly vulnerable to the identified (positive and negative) modifiable factors. Tracts often implicated include the anterior corpus callosum, uncinate fasciculus, superior longitudinal fasciculus, inferior fronto-occipital fasciculus, cingulum and (anterior) thalamic radiation (Fig. 3). The integrity of these tracts is known to decline with age and most of these tracts would be described as late-myelinating (e.g., genu of corpus callosum, association fibers, and thalamic radiation [Benitez et al., 2018, Brickman et al., 2012]), which may indicate a heightened vulnerability of these tracts to modifiable factors over and above effects of age. Moreover, most of these tracts have been cited to play a role in various cognitive processes (Madden et al., 2012), suggesting a pathway whereby changing exposure to the various factors could possibly prevent or slow age-related WM breakdown, cognitive decline, and AD risk (Gold et al., 2012). It is important to note, however, that modification of factors may have differential effects on WM, depending for instance, on an individual's sex or genetic makeup.

### 6.2 Methodological considerations

Substantial heterogeneity exists between studies included in this review, particularly in their designs, DTI acquisition and analysis methods. Most studies were cross-sectional in nature, and while many corrected for one or more confounding variables (e.g., age and sex), causality cannot be inferred. Furthermore, samples varied in their size and age range. For instance, cross-sectional studies investigating the relationship of hypertension with WM were well-sampled (N > 100 in 11 of 11 studies included in this review), whereas those of smoking were small (N > 100 in 2 of 8 included studies). Similarly, studies that focused on the relationship of sleep disturbances, social isolation, and meditation with WM integrity mainly included young adults, which limits their generalizability and warrant further study in older populations.

Although studies have shown that an advantage of DTI is its higher sensitivity to WM impairments compared with conventional MR methods (e.g., white matter hyperintensities [WMH]), the variety of ways in which DTI is acquired and analyzed across studies limits the extent to which findings can be compared. Although DTI measurements generally exhibit high reproducibility (Liu et al., 2014), especially for large WM tracts (Palacios et al., 2017), heterogeneity in acquisition parameters including the field strength, b-value(s), echo time, and number of diffusion-encoding directions, can result in variability and accuracy of DTI metrics across studies (Helmer et al., 2016, Liu et al., 2014). However, another study reported low intersite variation in DTI metrics and generally high reproducibility for large WM tracts, but more variability for the smaller tracts. Of the 31 studies included in Table 1, Table 2 in this review, field strength was most commonly 3T (21 studies), the b-value was most commonly 1000 s/mm^2^ (range: 700–1000 s/mm^2^ for a single b-value, 2 studies included 2 b-values: 1000 and 2000 s/mm^2^, and 2 studies did not report b-values); echo time and the number of diffusion-encoding directions ranged between 55 and 113 (median 87 ms) and 6 and 100 (median 37.5 directions), respectively. Further harmonization of acquisition protocols across studies and sites is warranted, particularly in light of recent multicentre trials. Freely available imaging protocols of big data neuroimaging initiatives such as the Human Connectome Project (Van Essen et al., 2013) or UK Biobank (Miller et al., 2016) could serve as templates and aid researchers who are in the process of deciding their imaging parameters.

Studies included in this review also varied in their DTI analysis methods. For instance, ROI approaches require specific tracts to be specified a priori—often based on previous research or hypotheses—and these have been used extensively for studies into hypertension and obesity. Even though replication of results is a good scientific practice, a focus on certain tracts only may bias tract-specific findings. Studies using whole-brain, voxelwise approaches may help reduce this bias, and could strengthen the evidence for tract-specific changes if findings were to be replicated.

In addition, differential inclusion of diffusivity metrics further limits a comparison of findings across studies. All studies included in this review reported the relationship between a modifiable factor and FA, fewer studies included MD, and only half of the studies also included RD and AxD. Although the biological interpretation of diffusivity metrics is still under debate, it is advisable to include AxD and RD given that these metrics may be more sensitive to WM changes than FA (Bennett and Madden, 2014) and can aid the interpretation of results.

Finally, the included studies varied in whether they controlled for the presence of comorbidities, differences in ethnicity and/or WMHs. Despite well-described relationships between risk factors (e.g., between hypertension and obesity [Ryan et al., 2017]), not all studies adjusted their analyses for the presence of comorbid factors, possibly biasing the findings. Furthermore, cardiovascular risk factors are more prevalent among certain ethnic groups, and some findings suggest that racial differences exist in gray and WM integrity (Liu et al., 2015), yet only few studies have taken racial differences into account (e.g., Hannawi et al., 2018, Rosano et al., 2015). Although a small number of studies limited their analyses to normal-appearing WM by masking out the WMH or corrected for WMHs in their analyses, a great proportion did not take WMHs into account (>50% of relevant studies in Table 1, Table 2). The presence of WMH has an effect on DTI metrics by reducing FA and increasing MD in affected regions; controlling for WMH in the analysis may increase power and reduce bias (Svärd et al., 2017).

### 6.3 Future directions

All of the included modifiable factors have a clear link with cognitive impairment and dementia (Livingston et al., 2017, Russell-Williams et al., 2018, Yaffe et al., 2016). Although it was outside the scope of this review to discuss in detail the relationship with cognitive performance, several studies have suggested that WM integrity may mediate the relationship between each factor and cognition. For instance, in cross-sectional studies, WM integrity was shown to mediate the relationship between cardiovascular fitness and working memory (Oberlin et al., 2016), between obesity and executive functions (Zhang et al., 2018), and between dietary nutrient intake and memory, language, and executive functions (Gu et al., 2016). In an interventional setting, exercise-induced FA increases were not related to improvements in working memory (Voss et al., 2013), whereas memory training induced WM changes were related to memory improvements (de Lange et al., 2017, Engvig et al., 2012). Further examining the possible mediating role of WM integrity in such relationships would be of great interest to the field.

While DTI is sensitive to changes in WM microstructure, it lacks specificity to specific features of WM tissue. Recently, more advanced diffusion MR modeling techniques have been developed (Alexander et al., 2017, Jelescu and Budde, 2017, Novikov et al., 2018a, Novikov et al., 2018b) that may provide additional information about tissue microstructure (see also Supplementary Material). The diffusion-based techniques are not without limitations (Novikov et al., 2018b), but allow one to link diffusion MR signals to parameters that characterize tissue properties, such as the intravoxel fiber dispersion or axonal volume fraction. Recently, one such method, White Matter Tract Integrity, has shown changes in extra-axonal diffusivities with increasing age, suggesting that myelin breakdown may be driving age-related WM breakdown (Benitez et al., 2018). White Matter Tract Integrity has been validated and has shown great promise for clinical applications (Jelescu and Budde, 2017), but has, to the best of our knowledge, not been used to study modifiable factors. By contrast, another biophysical model, neurite orientation dispersion and density estimation, has shown that hypertension is related to lower density of WM axons (Suzuki et al., 2017). Several other diffusion models have been developed that could further help interpret different biological mechanisms (Jelescu and Budde, 2017). Moreover, other nondiffusion methods may also be used to study microstructural features, such as magnetization transfer for myelin. Future studies should consider using novel biophysical models to explore WM tissue microstructure, as well as combining such techniques with other modalities (Alexander et al., 2017) to increase our understanding of the underlying biological changes with modification of risk factors.

For some modifiable factors, the field is arguably reaching a turning point at which studies can begin to play a greater role in informing public health conversations regarding why targeting a modifiable factor is recommended. While, historically, the neuroimaging literature has contributed minimally to guidelines on hypertension (James et al., 2014), physical activity (“2008 Physical Activity Guidelines for Americans,” 2008), and sleep (Hirshkowitz et al., 2015), the recent Global Council on Brain Health Expert Consensus on Physical Activity stated that “Based on randomized controlled trials, people who participate in purposeful exercise show beneficial changes in brain structure and function” (Global Council on Brain Health, 2016). For the DTI field to impact on initiatives such as the Global Council on Brain Health, careful intervention studies that examine changes in WM microstructure on modification of a factor are needed to strengthen the evidence-base around causality.

The extent to which the DTI literature can inform public health conversations regarding how a modifiable factor should be targeted (e.g., by indicating the minimum amount of sleep recommended for brain health; the optimal frequency, intensity, and type of physical activity) is currently limited. However, the advent of studies such as UK Biobank (Miller et al., 2016), which will scan 100,000 participants, and data sharing initiatives such as Dementia Platforms UK (“Dementias platform UK,” n.d.), mean that there is great potential for harmonization of DTI measures across studies. Meta-analyses could further help synthesize findings (Gurevitch et al., 2018) to inform public health discourse and give direction to research. However, to the best of our knowledge, a meta-analysis related to DTI has so far only been published for late-life depression (Wen et al., 2014).

Finally, although examining modifiable factors independently is vital to elucidating mechanistic pathways and establishing dose-response relationships, it is likely the combination of multiple factors that determines the integrity of the WM in older adults. Many factors coexist (Livingston et al., 2017, Wang et al., 2015), and while most studies aimed to examine the direct effects of individual factors, usually by treating (some) of the other factors as nuisance variables, various approaches have been used to examine the direct, indirect, and/or joint effects of multiple factors. Some studies, for instance, recruit participants based on the presence of one or multiple factors and compare WM integrity between the groups. This approach has been used to study the relative contribution of obesity and diabetes to WM integrity (van Bloemendaal et al., 2016); by comparing obese diabetic, obese, and lean participants, it was shown that reduced WM integrity in obese diabetics may be largely explained by BMI rather than by the presence of diabetes. An alternative approach is to use statistical models, such as mediation analysis by regression or structural equation modeling (VanderWeele, 2016), to examine the direct and indirect relationships between factors. While the use of mediation models in cross-sectional studies relies on certain assumptions (VanderWeele, 2016), this approach has been used to show that obesity may affect WM integrity directly, as well as indirectly through elevated blood pressure (Allen et al., 2016). A third approach is to examine the joint relationship of the presence of several factors with WM integrity. Using this approach, it has been demonstrated that co-occurrence of vascular risk factors is associated with greater decline of WM integrity (i.e., lower FA values) (Cox et al., 2019, Maillard et al., 2015, Wang et al., 2015, Williams et al., 2019) and that hypertension, smoking, and diabetes each have unique contributions to lower WM integrity (Cox et al., 2019). Observational studies that assess multiple risk and protective factors will allow a greater understanding of how factors interact, and their relative importance. Indeed, as effect sizes have often not been reported and may be small (Sexton et al., 2017), it is multidomain interventions that may hold most promise for studies with the primary aim of improving WM integrity in aging populations.

## 7 Conclusion

Decline in WM structure is a key hallmark of the aging brain, and an established risk factor for dementia, stroke, and mortality (Debette and Markus, 2010). Various factors have been shown to modulate the integrity of the WM during the aging process. Vascular risk factors, such as hypertension, diabetes, obesity, and smoking, as well as depressive symptoms, hearing loss, social isolation, and sleep disturbances, have been related to a loss of WM integrity. Conversely, a higher level of physical activity, a healthy diet, cognitive training, and meditation, appear to protect WM integrity. Encouragingly, preliminary evidence from cross-sectional and intervention studies suggests that successful modification of factors may impact on WM integrity, and this literature may contribute to public health initiatives going forward.

## Disclosure

C.E.S. reports receiving consulting fees from Jazz Pharmaceuticals.

## References

[bib1] Abe O., Takao H., Gonoi W., Sasaki H., Murakami M., Kabasawa H., Kawaguchi H., Goto M., Yamada H., Yamasue H., Kasai K., Aoki S., Ohtomo K. (2010). Voxel-based analysis of the diffusion tensor. Neuroradiology.

[bib2] Alexander D.C., Dyrby T.B., Nilsson M., Zhang H. (2017). Imaging brain microstructure with diffusion MRI: practicality and applications. NMR Biomed..

[bib3] Allan C.L., Sexton C.E., Filippini N., Topiwala A., Mahmood A., Zsoldos E.E., Singh-Manoux A., Shipley M.J., Kivimaki M., Mackay C.E., Ebmeier K.P. (2016). Sub-threshold depressive symptoms and brain structure: a magnetic resonance imaging study within the Whitehall II cohort. J. Affect. Disord..

[bib4] Allen B., Muldoon M.F., Gianaros P.J., Jennings J.R. (2016). Higher blood pressure partially links greater adiposity to reduced brain white matter integrity. Am. J. Hypertens..

[bib5] Amlien I.K., Fjell A.M. (2014). Diffusion tensor imaging of white matter degeneration in Alzheimer’s disease and mild cognitive impairment. Neuroscience.

[bib6] Antonenko D., Külzow N., Cesarz M.E., Schindler K., Grittner U., Flöel A. (2016). Hippocampal pathway plasticity is associated with the ability to form novel memories in older adults. Front. Aging Neurosci..

[bib7] Arenaza-Urquijo E.M., Wirth M., Chételat G. (2015). Cognitive reserve and lifestyle: moving towards preclinical Alzheimer’s disease. Front. Aging Neurosci..

[bib8] Baeza-Loya S., Velasquez K.M., Molfese D.L., Viswanath H., Curtis K.N., Thompson-Lake D.G., Baldwin P.R., Ellmore T.M., De La Garza R., Salas R. (2016). Anterior cingulum white matter is altered in tobacco smokers. Am. J. Addict..

[bib9] Baillet M., Dilharreguy B., Pérès K., Dartigues J.F., Mayo W., Catheline G. (2017). Activity/rest cycle and disturbances of structural backbone of cerebral networks in aging. Neuroimage.

[bib10] Barnes D.E., Yaffe K. (2011). The projected effect of risk factor reduction on Alzheimer’s disease prevalence. Lancet Neurol..

[bib11] Bartrés-Faz D., Arenaza-Urquijo E.M. (2011). Structural and functional imaging correlates of cognitive and brain reserve hypotheses in healthy and pathological aging. Brain Topogr..

[bib12] Bartzokis G. (2004). Age-related myelin breakdown: a developmental model of cognitive decline and Alzheimer’s disease. Neurobiol. Aging.

[bib13] Beaulieu C., Johansen-Berg H., Behrens T.E. (2009). The biological basis of diffusion anisotropy. Diffusion MRI: From Quantitative Measurement to In-Vivo Neuroanatomy.

[bib14] Bender A.R., Raz N. (2015). Normal-appearing cerebral white matter in healthy adults: mean change over two years and individual differences in change. Neurobiol. Aging.

[bib15] Bender A.R., Völkle M.C., Raz N. (2016). Differential aging of cerebral white matter in middle-aged and older adults: a seven-year follow-up. Neuroimage.

[bib16] Benitez A., Jensen J.H., Falangola M.F., Nietert P.J., Helpern J.A. (2018). Modeling white matter tract integrity in aging with diffusional kurtosis imaging. Neurobiol. Aging.

[bib17] Bennett I.J., Madden D.J. (2014). Disconnected aging: cerebral white matter integrity and age-related differences in cognition. Neuroscience.

[bib18] Best J.R., Rosano C., Aizenstein H.J., Tian Q., Boudreau R.M., Ayonayon H.N., Satterfield S., Simonsick E.M., Studenski S., Yaffe K., Liu-Ambrose T. (2017). Long-term changes in time spent walking and subsequent cognitive and structural brain changes in older adults. Neurobiol. Aging.

[bib19] Bettcher B.M., Walsh C.M., Watson C., Miller J.W., Green R., Patel N., Miller B.L., Neuhaus J., Yaffe K., Kramer J.H. (2013). Body mass and white matter integrity: the influence of vascular and inflammatory markers. PLoS One.

[bib20] Biessels G.J., Reijmer Y.D. (2014). Brain changes underlying cognitive dysfunction in diabetes: what can we learn from MRI?. Diabetes.

[bib21] Birdsill A.C., Oleson S., Kaur S., Pasha E., Ireton A., Tanaka H., Haley A. (2017). Abdominal obesity and white matter microstructure in midlife. Hum. Brain Mapp..

[bib22] Boekel W., Hsieh S. (2018). Cross-sectional white matter microstructure differences in age and trait mindfulness. PLoS One.

[bib23] Bolzenius J.D., Laidlaw D.H., Cabeen R.P., Conturo T.E., McMichael A.R., Lane E.M., Heaps J.M., Salminen L.E., Baker L.M., Scott S.E., Cooley S.A., Gunstad J., Paul R.H. (2015). Brain structure and cognitive correlates of body mass index in healthy older adults. Behav. Brain Res..

[bib24] Brickman A.M., Meier I.B., Korgaonkar M.S., Provenzano F.A., Grieve S.M., Siedlecki K.L., Wasserman B.T., Williams L.M., Zimmerman M.E. (2012). Testing the white matter retrogenesis hypothesis of cognitive aging. Neurobiol. Aging.

[bib25] Burzynska A.Z., Wong C.N., Chaddock-Heyman L., Olson E.A., Gothe N.P., Knecht A., Voss M.W., McAuley E., Kramer A.F. (2015). White matter integrity, hippocampal volume, and cognitive performance of a world-famous nonagenarian track-and-field athlete. Neurocase.

[bib26] Burzynska A.Z., Jiao Y., Knecht A.M., Fanning J., Awick E.A., Chen T., Gothe N., Voss M.W., McAuley E., Kramer A.F. (2017). White matter integrity declined over 6-months, but dance intervention improved integrity of the Fornix of older adults. Front. Aging Neurosci..

[bib27] Cao X., Yao Y., Li T., Cheng Y., Feng W., Shen Y., Li Q., Jiang L., Wu W., Wang J., Sheng J., Feng J., Li C. (2016). The impact of cognitive training on cerebral white matter in community-dwelling elderly: one-year prospective longitudinal diffusion tensor imaging study. Sci. Rep..

[bib28] Carnevale L., D’Angelosante V., Landolfi A., Grillea G., Selvetella G., Storto M., Lembo G., Carnevale D. (2018). Brain MRI fiber-tracking reveals white matter alterations in hypertensive patients without damage at conventional neuroimaging. Cardiovasc. Res..

[bib29] Castronovo V., Scifo P., Castellano A., Aloia M.S., Iadanza A., Marelli S., Cappa S.F., Strambi L.F., Falini A. (2014). White matter integrity in obstructive sleep apnea before and after treatment. Sleep.

[bib30] Chapman S.B., Aslan S., Spence J.S., Hart J.J., Bartz E.K., Didehbani N., Keebler M.W., Gardner C.M., Strain J.F., Defina L.F., Lu H. (2015). Neural mechanisms of brain plasticity with complex cognitive training in healthy seniors. Cereb. Cortex.

[bib31] Chen H.L., Lu C.H., Lin H.C., Chen P.C., Chou K.H., Lin W.M., Tsai N.W., Su Y.J., Friedman M., Lin C.P., Lin W.C. (2015). White matter damage and systemic inflammation in obstructive sleep apnea. Sleep.

[bib32] Clerx L., Visser P.J., Verhey F., Aalten P. (2012). New MRI markers for alzheimer’s disease: a meta-analysis of diffusion tensor imaging and a comparison with medial temporal lobe measurements. J. Alzheimers Dis..

[bib33] Cox S.R., Ritchie S.J., Tucker-Drob E.M., Liewald D.C., Hagenaars S.P., Davies G., Wardlaw J.M., Gale C.R., Bastin M.E., Deary I.J. (2016). Ageing and brain white matter structure in 3,513 UK Biobank participants. Nat. Commun..

[bib34] Cox S.R., Lyall D.M., Ritchie S.J., Bastin M.E., Harris M.A., Buchanan C.R., Fawns-Ritchie C., Barbu M.C., de Nooij L., Reus L.M., Alloza C., Shen X., Neilson E., Alderson H.L., Hunter S., Liewald D.C., Whalley H.C., McIntosh A.M., Lawrie S.J., Pell J.P., Tucker-Drob E.M., Wardlaw J.M., Gale C.R., Deary I.J. (2019). Associations between vascular risk factors and brain MRI indices in UK Biobank. Eur. Heart J..

[bib35] de Groot M., Ikram M.A., Akoudad S., Krestin G.P., Hofman A., Van Der Lugt A., Niessen W.J., Vernooij M.W. (2015). Tract-specific white matter degeneration in aging: the Rotterdam study. Alzheimers Dement..

[bib36] de Groot M., Cremers L.G., Ikram M.A., Hofman A., Krestin G.P., van der Lugt A., Niessen W.J., Vernooij M.W. (2016). White matter degeneration with aging: longitudinal diffusion MR imaging analysis. Radiology.

[bib37] de Lange A.G., Bråthen A.C.S., Grydeland H., Sexton C., Johansen-Berg H., Andersson J.L., Rohani D.A., Nyberg L., Fjell A.M., Walhovd K.B. (2016). White-matter integrity as a marker for cognitive plasticity in aging. Neurobiol. Aging.

[bib38] de Lange A.G., Bråthen A.C.S., Rohani D.A., Grydeland H., Fjell A.M., Walhovd K.B. (2017). The effects of memory training on behavioral and microstructural plasticity in young and older adults. Hum. Brain Mapp..

[bib39] Debette S., Markus H.S. (2010). The clinical importance of white matter hyperintensities on brain magnetic resonance imaging : systematic review and meta-analysis. BMJ.

[bib40] Elvsåshagen T., Norbom L.B., Pedersen P.Ø., Quraishi S.H., Bjørnerud A., Malt U.F., Groote I.R., Westlye L.T. (2015). Widespread changes in white matter microstructure after a day of waking and sleep deprivation. PLoS One.

[bib41] Engvig A., Fjell A.M., Westlye L.T., Moberget T., Sundseth Ø., Larsen V.A., Walhovd K.B. (2012). Memory training impacts short-term changes in aging white matter: a Longitudinal Diffusion Tensor Imaging Study. Hum. Brain Mapp..

[bib42] Falvey C.M., Rosano C., Simonsick E.M., Harris T., Strotmeyer E.S., Satterfield S., Yaffe K. (2013). Macro- and microstructural magnetic resonance imaging indices associated with diabetes among community-dwelling older adults. Diabetes Care.

[bib43] Filley C.M., Fields R.D. (2016). White matter and cognition: making the connection. J. Neurophysiol..

[bib44] Fissler P., Müller H., Küster O.C., Laptinskaya D., Thurm F., Woll A., Elbert T., Kassubek J., Von Arnim C.A., Kolassa I. (2017). No evidence that short-term cognitive or physical training programs or lifestyles are related to changes in white matter integrity in older adults at risk of dementia. Front. Hum. Neurosci..

[bib45] Fotuhi M., Do D., Jack C. (2012). Modifiable factors that alter the size of the hippocampus with ageing. Nat. Rev. Neurol..

[bib46] Gadie A., Shafto M., Leng Y., Kievit R.A. (2017). How are age-related differences in sleep quality associated with health outcomes? An epidemiological investigation in a UK cohort of 2406 adults. BMJ Open.

[bib47] Global Council on Brain Health (2016). The Brain-Body Connection: GCBH Recommendations on Physical Activity and Brain Health. http://www.GlobalCouncilOnBrainHealth.org.

[bib48] Gogliettino A.R., Potenza M.N., Yip S.W. (2016). White matter development and tobacco smoking in young adults: a systematic review with recommendations for future research. Drug Alcohol Depend..

[bib49] Gold B.T., Johnson N.F., Powell D.K., Smith C.D. (2012). White matter integrity and vulnerability to Alzheimer’s disease: preliminary findings and future directions. Biochim. Biophys. Acta.

[bib50] Gons R.A., Van Norden A.G., De Laat K.F., Van Oudheusden L.J., Van Uden I.W., Zwiers M.P., Norris D.G., De Leeuw F.E. (2011). Cigarette smoking is associated with reduced microstructural integrity of cerebral white matter. Brain.

[bib51] Gons R.A., Van Oudheusden L.J., De Laat K.F., Van Norden A.G., Van Uden I.W., Norris D.G., Zwiers M.P., Van Dijk E., De Leeuw F.E. (2012). Hypertension is related to the microstructure of the corpus callosum: the RUN DMC study. J. Alzheimers Dis..

[bib52] Gons R.A., Tuladhar A.M., de Laat K.F., van Norden A.G., van Dijk E.J., Norris D.G., Zwiers M.P., de Leeuw F.E. (2013). Physical activity is related to the structural integrity of cerebral white matter. Neurology.

[bib53] Gu Y., Vorburger R.S., Gazes Y., Habeck C.G., Stern Y., Luchsinger J.A., Manly J.J., Schupf N., Mayeux R., Brickman A.M. (2016). White matter integrity as a mediator in the relationship between dietary nutrients and cognition in the elderly. Ann. Neurol..

[bib54] Gurevitch J., Koricheva J., Nakagawa S., Stewart G. (2018). Review Meta-analysis and the science of research synthesis. Nature.

[bib55] Guttmann C.R.G., Jolesz F.A., Kikinis R., Killiany R.J., Moss M.B., Sandor T., Albert M.S. (1998). White matter changes with normal aging. Neurology.

[bib56] Hannawi Y., Yanek L.R., Kral B.G., Vaidya D., Becker L.C., Becker D.M., Nyquist P.A. (2018). Hypertension is associated with white matter disruption in apparently healthy middle-aged individuals. Am. J. Neuroradiol..

[bib57] Hayakawa Y.K., Sasaki H., Takao H., Mori H., Hayashi N., Kunimatsu A., Aoki S., Ohtomo K. (2013). Structural brain abnormalities in women with subclinical depression, as revealed by voxel-based morphometry and diffusion tensor imaging. J. Affect. Disord..

[bib58] Hayakawa Y.K., Sasaki H., Takao H., Hayashi N., Kunimatsu A., Ohtomo K., Aoki S. (2014). Depressive symptoms and neuroanatomical structures in community-dwelling women: a combined voxel-based morphometry and diffusion tensor imaging study with tract-based spatial statistics. Neuroimage Clin..

[bib59] Hayes S.M., Salat D.H., Forman D.E., Sperling R.A., Verfaellie M. (2015). Cardiorespiratory fitness is associated with white matter integrity in aging. Ann. Clin. Transl. Neurol..

[bib60] Helmer K.G., Chou M.C., Preciado R.I., Gimi B., Rollins N.K., Song A., Turner J., Mori S. (2016). Multi-site Study of Diffusion Metric Variability: Characterizing the Effects of Site, Vendor, Field Strength, and Echo Time Using the Histogram Distance. Proc SPIE Int Soc Opt Eng..

[bib61] Hirshkowitz M., Whiton K., Albert S.M., Alessi C., Bruni O., Doncarlos L., Hazen N., Herman J., Katz E.S., Kheirandish-gozal L., Neubauer D.N., Donnell A.E.O., Ohayon M., Peever J., Rawding R., Sachdeva R.C., Setters B., Vitiello M.V., Ware J.C., Hillard P.J.A. (2015). National Sleep Foundation ’ s sleep time duration recommendations : methodology and results summary. Sleep Heal..

[bib62] Hölzel B.K., Brunsch V., Gard T., Greve D.N., Koch K., Sorg C., Lazar S.W., Milad M.R. (2016). Mindfulness-based stress reduction, fear conditioning, and the uncinate fasciculus: a pilot study. Front. Behav. Neurosci..

[bib63] Hoogenboom W.S., Marder T.J., Flores V.L., Huisman S., Eaton H.P., Schneiderman J.S., Bolo N.R., Simonson D.C., Jacobson A.M., Kubicki M., Shenton M.E., Musen G. (2014). Cerebral white matter integrity and resting-state functional connectivity in middle-aged patients with type 2 diabetes. Diabetes.

[bib64] Hsu J.L., Chen Y.L., Leu J.G., Jaw F.S., Lee C.H., Tsai Y.F., Hsu C.Y., Bai C.H., Leemans A. (2012). Microstructural white matter abnormalities in type 2 diabetes mellitus: a diffusion tensor imaging study. Neuroimage.

[bib65] Hudkins M., O’Neill J., Tobias M.C., Bartzokis G., London E.D. (2012). Cigarette smoking and white matter microstructure. Psychopharmacology (Berl).

[bib66] James P.A., Oparil S., Carter B.L., Cushman W.C., Dennison-Himmelfarb C., Handler J., Lackland D.T., LeFevre M.L., MacKenzie T.D., Ogedegbe O., Smith S.C., Svetkey L.P., Taler S.J., Townsend R.R., Wright J.T., Narva A.S., Ortiz E. (2014). 2014 evidence-based guideline for the management of high blood pressure in adults report from the panel members appointed to the eighth joint national committee (JNC 8). JAMA.

[bib67] Jbabdi S., Johansen-Berg H. (2011). Tractography: where do we go from here?. Brain Connect..

[bib68] Jbabdi S., Sotiropoulos S.N., Haber S.N., Van Essen D.C., Behrens T.E., Van Essen D.C., Behrens T.E. (2015). Measuring macroscopic brain connections in vivo. Nat. Neurosci..

[bib69] Jelescu I.O., Budde M.D. (2017). Design and validation of diffusion MRI models of white matter. Front. Phys..

[bib70] Jones D.K., Symms M.R., Cercignani M., Howard R.J. (2005). The effect of filter size on VBM analyses of DT-MRI data. Neuroimage.

[bib71] Jones D.K., Knösche T.R., Turner R. (2013). White matter integrity, fiber count, and other fallacies: the do’s and don’ts of diffusion MRI. Neuroimage.

[bib72] Kang D.H., Jo H.J., Jung W.H., Kim S.H., Jung Y.H., Choi C.H., Lee U.S., An S.C., Jang J.H., Kwon J.S. (2013). The effect of meditation on brain structure: cortical thickness mapping and diffusion tensor imaging. Soc. Cogn. Affect. Neurosci..

[bib73] Karlsson H.K., Tuulari J.J., Hirvonen J., Lepomäki V., Parkkola R., Hiltunen J., Hannukainen J.C., Soinio M., Pham T., Salminen P., Nuutila P., Nummenmaa L. (2013). Obesity is associated with white matter atrophy: a combined diffusion tensor imaging and voxel-based morphometric study. Obesity.

[bib74] Khalsa S., Hale J.R., Goldstone A., Wilson R.S., Mayhew S.D., Bagary M., Bagshaw A.P. (2017). Habitual sleep durations and subjective sleep quality predict white matter differences in the human brain. Neurobiol. Sleep Circadian Rhythm..

[bib75] Kocevska D., Cremers L.G.M., Lysen T.S., Luik A.I., Ikram M.A., Vernooij M.W., Tiemeier H. (2019). Sleep complaints and cerebral white matter: a prospective bidirectional study. J. Psychiatr. Res..

[bib76] Kochunov P., Williamson D.E., Lancaster J., Fox P., Cornell J., Blangero J., Glahn D.C. (2012). Fractional anisotropy of water diffusion in cerebral white matter across the lifespan. Neurobiol. Aging.

[bib77] Kochunov P., Du X., Moran L.V., Sampath H., Andrea Wijtenburg S., Yang Y., Rowland L.M., Stein E.A., Elliot Hong L. (2013). Acute nicotine administration effects on fractional anisotropy of cerebral white matter and associated attention performance. Front. Pharmacol..

[bib78] Köhncke Y., Laukka E.J., Brehmer Y., Kalpouzos G., Li T.Q., Fratiglioni L., Bäckman L., Lövdén M. (2016). Three-year changes in leisure activities are associated with concurrent changes in white matter microstructure and perceptual speed in individuals aged 80 years and older. Neurobiol. Aging.

[bib79] Krafft C.E., Schaeffer D.J., Schwarz N.F., Chi L., Weinberger A.L., Pierce J.E., Rodrigue A.L., Allison J.D., Yanasak N.E., Liu T., Davis C.L., McDowell J.E. (2014). Improved frontoparietal white matter integrity in overweight children is associated with attendance at an after-school exercise program. Dev. Neurosci..

[bib80] Kullmann S., Schweizer F., Veit R., Fritsche A., Preissl H. (2015). Compromised white matter integrity in obesity. Obes. Rev..

[bib81] Kullmann S., Callaghan M.F., Heni M., Weiskopf N., Scheffler K., Häring H.U., Fritsche A., Veit R., Preissl H. (2016). Specific white matter tissue microstructure changes associated with obesity. Neuroimage.

[bib82] Lampit A., Hallock H., Suo C., Naismith S.L., Valenzuela M. (2015). Cognitive training-induced short-term functional and long-term structural plastic change is related to gains in global cognition in healthy older adults: a pilot study. Front. Aging Neurosci..

[bib83] Laneri D., Schuster V., Dietsche B., Jansen A., Ott U., Sommer J. (2016). Effects of long-term mindfulness meditation on Brain’s white matter microstructure and its aging. Front. Aging Neurosci..

[bib84] Le Bihan D., Johansen-Berg H. (2012). Diffusion MRI at 25: exploring brain tissue structure and function. Neuroimage.

[bib85] Lebel C., Gee M., Camicioli R., Wieler M., Martin W., Beaulieu C. (2012). Diffusion tensor imaging of white matter tract evolution over the lifespan. Neuroimage.

[bib86] Li S., Tian J., Bauer A., Huang R., Wen H., Li M., Wang T., Xia L., Jiang G. (2016). Reduced integrity of right lateralized white matter in patients with primary insomnia: a diffusion-tensor imaging study. Radiology.

[bib87] Lin F., Wu G., Zhu L., Lei H. (2013). Heavy smokers show abnormal microstructural integrity in the anterior corpus callosum: a diffusion tensor imaging study with tract-based spatial statistics. Drug Alcohol Depend..

[bib88] Liu X., Yang Y., Sun J., Yu G., Xu J., Niu C., Tian H., Lin P. (2014). Reproducibility of diffusion tensor imaging in normal subjects: an evaluation of different gradient sampling schemes and registration algorithm. Neuroradiology.

[bib89] Liu G., Allen B., Lopez O., Aizenstein H., Boudreau R., Newman A., Yaffe K., Kritchevsky S., Launer L., Satterfield S., Simonsick E., Rosano C. (2015). Racial differences in gray matter integrity by diffusion tensor in black and white octogenarians. Curr. Alzheimer Res..

[bib90] Livingston G., Sommerlad A., Orgeta V., Costafreda S.G., Huntley J., Ames D., Ballard C., Banerjee S., Burns A., Cohen-Mansfield J., Cooper C., Fox N., Gitlin L.N., Howard R., Kales H.C., Larson E.B., Ritchie K., Rockwood K., Sampson E.L., Samus Q., Schneider L.S., Selbæk G., Teri L., Mukadam N. (2017). Dementia prevention, intervention, and care. Lancet.

[bib91] Luders E., Clark K., Narr K.L., Toga A.W. (2011). Enhanced brain connectivity in long-term meditation practitioners. Neuroimage.

[bib92] Luders E., Phillips O.R., Clark K., Kurth F., Toga A.W., Narr K.L. (2012). Bridging the hemispheres in meditation: thicker callosal regions and enhanced fractional anisotropy (FA) in long-term practitioners. Neuroimage.

[bib93] Lyden H., Espinoza R.T., Pirnia T., Clark K., Joshi S.H., Leaver A.M., Woods R.P., Narr K.L. (2014). Electroconvulsive therapy mediates neuroplasticity of white matter microstructure in major depression. Transl. Psychiatry.

[bib94] Ma W., Li M., Gao F., Zhang X., Shi L., Yu L., Zhao B., Chen W., Wang G., Wang X. (2016). DTI analysis of presbycusis using voxel-based analysis. Am. J. Neuroradiol..

[bib95] Madden D.J., Bennett I.J., Burzynska A., Potter G.G., Chen N.K., Song A.W. (2012). Diffusion tensor imaging of cerebral white matter integrity in cognitive aging. Biochim. Biophys. Acta.

[bib96] Maillard P., Seshadri S., Beiser A., Himali J.J., Au R., Fletcher E., Carmichael O., Wolf P.A., DeCarli C. (2012). Effects of systolic blood pressure on white-matter integrity in young adults in the Framingham Heart Study: a cross-sectional study. Lancet Neurol..

[bib97] Maillard P., Carmichael O.T., Reed B., Mungas D., DeCarli C. (2015). Cooccurrence of vascular risk factors and late-life white-matter integrity changes. Neurobiol. Aging.

[bib98] Maillard P., Mitchell G.F., Himali J.J., Beiser A., Tsao C.W., Pase M.P., Satizabal C.L., Vasan R.S., Seshadri S., De Carli C. (2016). Effects of arterial stiffness on brain integrity in young adults from the framingham heart study. Stroke.

[bib99] Mander B.A., Zhu A.H., Lindquist J.R., Villeneuve S., Rao V., Lu B., Jared M., Ancoli-israel S., Jagust W., Walker M.P., Mander B.A., Zhu A.H., Lindquist J.R., Villeneuve S., Rao V. (2017). White matter structure in older adults moderates the benefit of sleep spindles on motor memory consolidation. J. Neurosci..

[bib100] Mcevoy L.K., Fennema-Notestine C., Eyler L.T., Franz C.E., Hagler D.J., Lyons M.J., Panizzon M.S., Rinker D.A., Dale A.M., Kremen W.S. (2015). Hypertension-related alterations in white matter microstructure detectable in middle age. Hypertension.

[bib101] McIntosh A.M., Bastin M.E., Luciano M., Muñoz Maniega S., Valdés Hernández M., Royle N.A., Hall J., Murray C., Lawrie S.M., Starr J.M., Wardlaw J.M., Deary I.J. (2013). Neuroticism , depressive symptoms and white-matter integrity in the Lothian Birth Cohort 1936. Psychol. Med..

[bib102] Meng J., Hao L., Wei D., Sun J., Li Y., Qiu J. (2017). BDNF Val66Met polymorphism modulates the effect of loneliness on white matter microstructure in young adults. Biol. Psychol..

[bib103] Miller K.L., Alfaro-Almagro F., Bangerter N.K., Thomas D.L., Yacoub E., Xu J., Bartsch A.J., Jbabdi S., Sotiropoulos S.N., Andersson J.L., Griffanti L., Douaud G., Okell T.W., Weale P., Dragonu I., Garratt S., Hudson S., Collins R., Jenkinson M., Matthews P.M., Smith S.M. (2016). Multimodal population brain imaging in the UK Biobank prospective epidemiological study. Nat. Neurosci..

[bib104] Molesworth T., Sheu L.K., Cohen S., Gianaros P.J., Verstynen T.D. (2015). Social network diversity and white matter microstructural integrity in humans. Soc. Cogn. Affect. Neurosci..

[bib105] Morris M.C., Tangney C.C., Wang Y., Sacks F.M., Barnes L.L., Bennett D.A., Aggarwal N.T. (2015). MIND diet slows cognitive decline with aging. Alzheimers Dement..

[bib106] Morris M.C., Tangney C.C., Wang Y., Sacks F.M., Bennett D.A., Aggarwal N.T. (2015). MIND diet associated with reduced incidence of Alzheimer’s disease. Alzheimers Dement..

[bib107] Murphy C. (2019). Olfactory and other sensory impairments in Alzheimer disease. Nat. Rev. Neurol..

[bib108] Nasrabady S.E., Rizvi B., Goldman J.E., Brickman A.M. (2018). White matter changes in Alzheimer’s disease: a focus on myelin and oligodendrocytes. Acta Neuropathol. Commun..

[bib109] Novikov D.S., Fieremans E., Jespersen S.N., Kiselev V.G. (2018). Quantifying brain microstructure with diffusion MRI: theory and parameter estimation. NMR Biomed..

[bib110] Novikov D.S., Kiselev V.G., Jespersen S.N. (2018). On modeling. Magn. Reson. Med..

[bib111] Oberlin L.E., Verstynen T.D., Burzynska A.Z., Voss M.W., Prakash R.S., Chaddock-Heyman L., Wong C., Fanning J., Awick E., Gothe N., Phillips S.M., Mailey E., Ehlers D., Olson E., Wojcicki T., McAuley E., Kramer A.F., Erickson K.I. (2016). White matter microstructure mediates the relationship between cardiorespiratory fitness and spatial working memory in older adults. Neuroimage.

[bib112] O’Donnell L.J., Pasternak O. (2015). Does diffusion MRI tell us anything about the white matter? An overview of methods and pitfalls. Schizophr. Res..

[bib113] Palacios E.M., Martin A.J., Boss M.A., Ezekiel F., Chang Y.S., Yuh E.L., Vassar M.J., Schnyer D.M., MacDonald C.L., Crawford K.L., Irimia A., Toga A.W., Mukherjee P. (2017). Toward precision and reproducibility of diffusion tensor imaging: a multicenter diffusion phantom and traveling volunteer study. Am. J. Neuroradiol..

[bib114] Papageorgiou I., Astrakas L.G., Xydis V., Alexiou G.A., Bargiotas P., Tzarouchi L., Zikou A.K., Kiortsis D.N., Argyropoulou M.I. (2017). Abnormalities of brain neural circuits related to obesity : a Diffusion Tensor Imaging study. Magn. Reson. Imaging.

[bib115] Pelletier A., Barul C., Féart C., Helmer C., Bernard C., Periot O., Dilharreguy B., Dartigues J.F., Allard M., Barberger-Gateau P., Catheline G., Samieri C. (2015). Mediterranean diet and preserved brain structural connectivity in older subjects. Alzheimers Dement..

[bib116] Peters A. (2002). The effects of normal aging on nerve fibers and neuroglia in the central nervous system. J. Neurocytol..

[bib118] Power M.C., Tingle J.V., Reid R.I., Huang J., Sharrett A.R., Coresh J., Griswold M., Kantarci K., Jack C.R., Knopman D., Gottesman R.F., Mosley T.H. (2017). Midlife and late-life vascular risk factors and white matter microstructural integrity: the atherosclerosis risk in communities neurocognitive study. J. Am. Heart Assoc..

[bib119] Prins N.D., Scheltens P. (2015). White matter hyperintensities, cognitive impairment and dementia: an update. Nat. Rev. Neurol..

[bib120] Profant O., Škoch A., Balogová Z., Tintěra J., Hlinka J., Syka J. (2014). Diffusion tensor imaging and MR morphometry of the central auditory pathway and auditory cortex in aging. Neuroscience.

[bib121] Reijmer Y.D., Brundel M., De Bresser J., Kappelle L.J., Leemans A., Biessels G.J. (2013). Microstructural white matter abnormalities and cognitive functioning in type 2 diabetes: a diffusion tensor imaging study. Diabetes Care.

[bib122] Repple J., Opel N., Meinert S., Redlich R., Hahn T., Winter N.R., Kaehler C., Emden D., Leenings R., Grotegerd D., Zaremba D., Bürger C., Förster K., Dohm K., Enneking V., Leehr E.J., Böhnlein J., Karliczek G., Heindel W., Kugel H., Bauer J., Arolt V., Dannlowski U. (2018). Elevated body-mass index is associated with reduced white matter integrity in two large independent cohorts. Psychoneuroendocrinology.

[bib123] Rigters S.C., Cremers L.G.M., Ikram M.A., van der Schroeff M.P., de Groot M., Roshchupkin G.V., Niessen W.J.N., Baatenburg de Jong R.J., Goedegebure A., Vernooij M.W. (2018). White-matter microstructure and hearing acuity in older adults: a population-based cross-sectional DTI study. Neurobiol. Aging.

[bib124] Rosano C., Abebe K.Z., Aizenstein H.J., Boudreau R., Jennings J.R., Venkatraman V., Harris T.B., Yaffe K., Satterfield S., Newman A.B. (2015). Longitudinal systolic blood pressure characteristics and integrity of white matter tracts in a cohort of very old black and white adults. Am. J. Hypertens..

[bib125] Russell-Williams J., Jaroudi W., Perich T., Hoscheidt S., El Haj M., Moustafa A.A. (2018). Mindfulness and meditation: treating cognitive impairment and reducing stress in dementia. Rev. Neurosci..

[bib126] Ryan L., Walther K. (2014). White matter integrity in older females is altered by increased body fat. Obesity.

[bib127] Ryan M., Kochunov P., Rowland L.M., Mitchell B.D., Wijtenburg S.A., Fieremans E., Veraart J., Novikov D.S., Du X., Adhikari B., Fisseha F., Bruce H., Chiappelli J., Sampath H., Ament S., O’Connell J., Shuldiner A.R., Hong L.E. (2017). Lipid metabolism, abdominal adiposity, and cerebral health in the amish. Obesity.

[bib128] Sabisz A., Naumczyk P., Marcinkowska A., Graff B., Gąsecki D., Glińska A., Witkowska M., Jankowska A., Konarzewska A., Kwela J., Jodzio K., Szurowska E., Narkiewicz K. (2019). Aging and hypertension – independent or intertwined white matter impairing factors? Insights from the quantitative diffusion tensor imaging. Front. Aging Neurosci..

[bib129] Sachdev P.S., Zhuang L., Braidy N., Wen W. (2013). Is Alzheimer’s a disease of the white matter?. Curr. Opin. Psychiatry.

[bib130] Salat D.H., Williams V.J., Leritz E.C., Schnyer D.M., Rudolph J.L., Lipsitz L.A., McGlinchey R.E., Milberg W.P. (2012). Inter-individual variation in blood pressure is associated with regional white matter integrity in generally healthy older adults. Neuroimage.

[bib131] Sampaio-Baptista C., Johansen-Berg H. (2017). White matter plasticity in the adult brain. Neuron.

[bib132] Savjani R.R., Velasquez K.M., Thompson-Lake D.G., Baldwin P.R., Eagleman D.M., De La Garza R., Salas R. (2014). Characterizing white matter changes in cigarette smokers via diffusion tensor imaging. Drug Alcohol Depend..

[bib133] Schaeffer D.J., Krafft C.E., Schwarz N.F., Chi L., Rodrigue A.L., Pierce J.E., Allison J.D., Yanasak N.E., Liu T., Davis C.L., McDowell J.E. (2014). An 8-month exercise intervention alters frontotemporal white matter integrity in overweight children. Psychophysiology.

[bib134] Sexton C.E., Kalu U.G., Filippini N., Mackay C.E., Ebmeier K.P. (2011). A meta-analysis of diffusion tensor imaging in mild cognitive impairment and Alzheimer’s disease. Neurobiol. Aging.

[bib135] Sexton C.E., Walhovd K., Storsve A.B., Tamnes C.K., Westlye L.T., Johansen-Berg H., Fjell A.M. (2014). Accelerated changes in white matter microstructure during ageing: a longitudinal diffusion tensor imaging study. J. Neurosci..

[bib136] Sexton C.E., Betts J.F., Demnitz N., Dawes H., Ebmeier K.P., Johansen-Berg H. (2016). A systematic review of MRI studies examining the relationship between physical fitness and activity and the white matter of the ageing brain. Neuroimage.

[bib137] Sexton C.E., Zsoldos E., Filippini N., Griffanti L., Winkler A., Mahmood A., Allan C.L., Topiwala A., Kyle S.D., Spiegelhalder K., Singh-Manoux A., Kivimaki M., Mackay C.E., Johansen-Berg H., Ebmeier K.P. (2017). Associations between self-reported sleep quality and white matter in community-dwelling older adults: a prospective cohort study. Hum. Brain Mapp..

[bib138] Sharma K., Trivedi R., Chandra S., Kaur P., Kumar P., Singh K., Dubey A.K., Khushu S. (2017). Enhanced white matter integrity in corpus callosum of long- term Brahmakumaris Rajayoga meditators. Brain Connect..

[bib139] Smith S.M., Jenkinson M., Johansen-Berg H., Rueckert D., Nichols T.E., Mackay C.E., Watkins K.E., Ciccarelli O., Cader M.Z., Matthews P.M., Behrens T.E. (2006). Tract-based spatial statistics: voxelwise analysis of multi-subject diffusion data. Neuroimage.

[bib140] Smith J.C., Lancaster M.A., Nielson K.A., Woodard J.L., Seidenberg M., Durgerian S., Sakaie K., Rao S.M. (2016). IInteractive effects of physical activity and APOE-e4 on white matter tract diffusivity in healthy elders. Neuroimage.

[bib141] Song S.K., Sun S.W., Ju W.K., Lin S.J., Cross A.H., Neufeld A.H. (2003). Diffusion tensor imaging detects and differentiates axon and myelin degeneration in mouse optic nerve after retinal ischemia. Neuroimage.

[bib142] Spieker E.A., Kochunov P., Rowland L.M., Sprooten E., Winkler A.M., Olvera R.L., Almasy L., Duggirala R., Fox P.T., Blangero J., Glahn D.C., Curran J.E. (2015). Shared genetic variance between obesity and white matter integrity in Mexican Americans. Front. Genet..

[bib143] Stanek K.M., Grieve S.M., Brickman A.M., Korgaonkar M.S., Paul R.H., Cohen R.A., Gunstad J.J. (2011). Obesity is associated with reduced white matter integrity in otherwise healthy adults. Obesity (Silver Spring).

[bib144] Stern Y. (2012). Cognitive reserve in ageing and Alzheimer’s disease. Lancet Neurol..

[bib145] Strenziok M., Parasuraman R., Clarke E., Cisler D.S., Thompson J.C., Greenwood P.M. (2014). Neurocognitive enhancement in older adults: comparison of three cognitive training tasks to test a hypothesis of training transfer in brain connectivity. Neuroimage.

[bib146] Sullivan E.V., Pfefferbaum A. (2006). Diffusion tensor imaging and aging. Neurosci. Biobehav. Rev..

[bib147] Sun S.W., Liang H.F., Trinkaus K., Cross A.H., Armstrong R.C., Song S.K. (2006). Noninvasive detection of cuprizone induced axonal damage and demyelination in the mouse corpus callosum. Magn. Reson. Med..

[bib148] Suri S., Topiwala A., Mackay C.E., Ebmeier K.P., Filippini N. (2014). Using structural and diffusion magnetic resonance imaging to differentiate the dementias. Curr. Neurol. Neurosci. Rep..

[bib149] Suzuki H., Gao H., Bai W., Evangelou E., Glocker B., Regan P.O., Elliott P., Matthews P.M. (2017). Abnormal brain white matter microstructure is associated with both pre-hypertension and hypertension. PLoS One.

[bib150] Svärd D., Nilsson M., Lampinen B., Lätt J., Sundgren P.C., Stomrud E., Minthon L., Hansson O., Van Westen D. (2017). The effect of white matter hyperintensities on statistical analysis of diffusion tensor imaging in cognitively healthy elderly and prodromal Alzheimer’s disease. PLoS One.

[bib151] Tan X., Fang P., An J., Lin H., Liang Y., Shen W., Leng X., Zhang C., Zheng Y., Qiu S. (2016). Micro-structural white matter abnormalities in type 2 diabetic patients: a DTI study using TBSS analysis. Neuroradiology.

[bib152] Tang Y.Y., Lu Q., Fan M., Yang Y., Posner M.I. (2012). Mechanisms of white matter changes induced by meditation. Proc. Natl. Acad. Sci.U. S. A..

[bib153] Tarabichi O., Kozin E.D., Kanumuri V.V., Barber S., Ghosh S., Sitek K.R., Reinshagen K., Herrmann B., Remenschneider A.K., Lee D.J. (2017). Diffusion tensor imaging of central auditory pathways in patients with sensorineural hearing loss: a systematic review. Otolaryngol. Head Neck Surg..

[bib154] Tian Y., Liang S., Yuan Z., Chen S., Xu P., Yao D. (2014). White matter structure in loneliness. Neuroreport.

[bib155] Tian Q., Glynn N.W., Erickson K.I., Aizenstein H.J., Simonsick E.M., Yaffe K., Harris T.B., Kritchevsky S.B., Boudreau R.M., Newman A.B., Lopez O.L., Saxton J., Rosano C. (2015). Objective measures of physical activity, white matter integrity and cognitive status in adults over age 80. Behav. Brain Res..

[bib156] Tournier J.D., Mori S., Leemans A. (2011). Diffusion tensor imaging and beyond. Magn. Reson. Med..

[bib157] Tudorascu D.L., Rosano C., Venkatraman V.K., MacCloud R.L., Harris T., Yaffe K., Newman A.B., Aizenstein H.J. (2014). Multimodal MRI markers support a model of small vessel ischemia for depressive symptoms in very old adults. Psychiatry Res. Neuroimaging.

[bib158] Umene-Nakano W., Yoshimura R., Kakeda S., Watanabe K., Hayashi K., Nishimura J., Takahashi H., Moriya J., Ide S., Ueda I., Hori H., Ikenouchi-Sugita A., Katsuki A., Atake K., Abe O., Korogi Y., Nakamura J. (2014). Abnormal white matter integrity in the corpus callosum among smokers: tract-based spatial statistics. PLoS One.

[bib117] United States Dept. of Health and Human Services (2008). 2008 Physical Activity Guidelines for Americans.

[bib159] van Bloemendaal L., Ijzerman R.G., ten Kulve J.S., Barkhof F., Diamant M., Veltman D.J., van Duinkerken E. (2016). Alterations in white matter volume and integrity in obesity and type 2 diabetes. Metab. Brain Dis..

[bib160] Van Essen D.C., Smith S.M., Barch D.M., Behrens T.E., Yacoub E. (2013). The Wu-minn human connectome Project: an overview. Neuroimage.

[bib161] VanderWeele T.J. (2016). Mediation analysis: a practitioner’s guide tyler. Annu. Rev. Public Health.

[bib162] Verkooijen S., Stevelink R., Abramovic L., Vinkers C.H., Opho A., Kahn R.S., Boks M.P., Van Haren N.E. (2017). The association of sleep and physical activity with integrity of white matter microstructure in bipolar disorder patients and healthy controls. Psychiatry Res. Neuroimaging.

[bib163] Viswanath H., Velasquez K.M., Thompson-Lake D.G.Y., Savjani R., Carter A.Q., Eagleman D., Baldwin P.R., De La Garza R., Salas R. (2015). Alterations in interhemispheric functional and anatomical connectivity are associated with tobacco smoking in humans. Front. Hum. Neurosci..

[bib164] Voss M.W., Heo S., Prakash R.S., Erickson K.I., Alves H., Chaddock L., Szabo A.N., Mailey E.L., Wójcicki T.R., White S.M., Gothe N., Mcauley E., Sutton B.P., Kramer A.F. (2013). The influence of aerobic fitness on cerebral white matter integrity and cognitive function in older adults: results of a one-year exercise intervention. Hum. Brain Mapp..

[bib165] Walhovd K.B., Johansen-Berg H., Káradóttir R.T. (2014). Unraveling the secrets of white matter - bridging the gap between cellular, animal and human imaging studies. Neuroscience.

[bib166] Wang T., Huang X., Huang P., Li D., Lv F., Zhang Y., Zhou L., Yang D., Xie P. (2013). Early-stage psychotherapy produces elevated frontal white matter integrity in adult major depressive disorder. PLoS One.

[bib167] Wang R., Fratiglioni L., Laukka E.J., Lövdén M., Keller L., Graff C. (2015). Effects of vascular risk factors and APOE e 4 on white matter integrity and cognitive decline. Neurology.

[bib168] Wen M.C., Steffens D.C., Chen M.K., Zainal N.H. (2014). Diffusion tensor imaging studies in late-life depression: systematic review and meta-analysis. Int. J. Geriatr. Psychiatry.

[bib169] Wheeler-Kingshott C.A., Cercignani M. (2009). About “axial” and “radial” diffusivities. Magn. Reson. Med..

[bib170] White W.B., Marfatia R., Schmidt J., Wakefield D.B., Kaplan R.F., Bohannon R.W., Hall C.B., Guttmann C.R., Moscufo N., Fellows D., Wolfson L. (2013). Intensive versus standard ambulatory blood pressure lowering to prevent functional decline in the elderly (INFINITY). Am. Heart J..

[bib171] Williams O.A., An Y., Beason-Held L., Huo Y., Ferrucci L., Landman B.A., Resnick S.M. (2019). Vascular burden and APOE ε4 are associated with white matter microstructural decline in cognitively normal older adults. Neuroimage.

[bib172] Williamson J.D., Pajewski N.M., Auchus A.P., Bryan R.N., Chelune G., Cheung A.K., Cleveland M.L., Coker L.H., Crowe M.G., Cushman W.C., Cutler J.A., Davatzikos C., Desiderio L., Erus G., Fine L.J., Gaussoin S.A., Harris D., Hsieh M.K., Johnson K.C., Kimmel P.L., Tamura M.K., Launer L.J., Lerner A.J., Lewis C.E., Martindale-Adams J., Moy C.S., Nasrallah I.M., Nichols L.O., Oparil S., Ogrocki P.K., Rahman M., Rapp S.R., Reboussin D.M., Rocco M.V., Sachs B.C., Sink K.M., Still C.H., Supiano M.A., Snyder J.K., Wadley V.G., Walker J., Weiner D.E., Whelton P.K., Wilson V.M., Woolard N., Wright J.T., Wright C.B. (2019). Effect of intensive vs standard blood pressure control on probable dementia: a randomized clinical trial. JAMA.

[bib173] Witte A.V., Kerti L., Hermannstädter H.M., Fiebach J.B., Schreiber S.J., Schuchardt J.P., Hahn A., Flöel A. (2014). Long-chain omega-3 fatty acids improve brain function and structure in older adults. Cereb. Cortex.

[bib174] Xiong X.Y., Sui X.Y., Xu X.Z., Zhang X.Q., Karaman M.M., Cai X.K., Anderson T.M., Zhu X.W., Wang X.J., Zhou X.X.J. (2016). A diffusion tensor imaging study on white matter abnormalities in patients with type 2 diabetes using tract-based spatial statistics. Am. J. Neuroradiol..

[bib175] Yaffe K., Falvey C.M., Hoang T. (2014). Connections between sleep and cognition in older adults. Lancet Neurol..

[bib176] Yaffe K., Nasrallah I., Hoang T.D., Lauderdale D.S., Knutson K.L., Carnethon M.R., Launer L.J., Lewis C.E., Sidney S. (2016). Sleep duration and white matter quality in middle-aged adults. Sleep.

[bib177] Yau P.L., Castro M.G., Tagani A., Tsui W.H., Convit A. (2012). Obesity and metabolic syndrome and functional and structural brain impairments in adolescence. Pediatrics.

[bib178] Yin R.H., Tan L., Liu Y., Wang W.Y., Wang H.F., Jiang T., Radua J., Zhang Y., Gao J., Canu E., Migliaccio R., Filippi M., Gorno-Tempini M.L., Yu J.T. (2015). Multimodal voxel-based meta-analysis of white matter abnormalities in alzheimer’s disease. J. Alzheimers Dis..

[bib179] Yu J., Lam C.L.M., Lee T.M.C. (2017). White matter microstructural abnormalities in amnestic mild cognitive impairment: a meta-analysis of whole-brain and ROI-based studies. Neurosci. Biobehav. Rev..

[bib180] Zamroziewicz M.K., Paul E.J., Zwilling C.E., Barbey A.K. (2018). Predictors of memory in healthy Aging : polyunsaturated fatty acid balance and fornix white matter integrity. Aging Dis..

[bib181] Zatorre R.J., Fields R.D., Johansen-berg H. (2012). Plasticity in gray and white : neuroimaging changes in brain structure during learning. Nat. Neurosci..

[bib182] Zhang J., Wang Y., Wang J., Zhou X., Shu N., Wang Y., Zhang Z. (2014). White matter integrity disruptions associated with cognitive impairments in type 2 diabetic patients. Diabetes.

[bib183] Zhang Y.E., Ji G., Xu M., Cai W., Zhu Q., Qian L., Zhang Y.E., Yuan K., Liu J., Li Q., Cui G., Wang H., Zhao Q., Wu K., Fan D., Gold M.S., Tian J., Tomasi D., Liu Y., Nie Y., Wang G.-J. (2016). Recovery of brain structural abnormalities in morbidly obese patients after bariatric surgery. Int. J. Obes. (Lond)..

[bib184] Zhang R., Beyer F., Lampe L., Luck T., Riedel-Heller S.G., Loeffler M., Schroeter M.L., Stumvoll M., Villringer A., Witte A.V. (2018). White matter microstructural variability mediates the relation between obesity and cognition in healthy adults. Neuroimage.

