# Supplementary material for: Associations between modifiable risk factors and white matter of the aging brain: insights from diffusion tensor imaging studies
Source: Neurobiol Aging. 2019 Aug;80:56–70. doi: 10.1016/j.neurobiolaging.2019.04.006 (PMC6683729; doi:10.1016/j.neurobiolaging.2019.04.006)
Supplement: Supplement [file mmc1.docx]

Supplementary material for:

**Associations between modifiable risk factors and white matter of the aging brain: Insights from diffusion tensor imaging studies**Thomas M. Wassenaar^a^ , Kristine Yaffe^b^ , Ysbrand D. van der Werf^c^ , Claire E. Sexton^d,e^ *

^a^ Wellcome Centre for Integrative Neuroscience, FMRIB Centre, Nuffield Department of Clinical Neurosciences, University of Oxford, John Radcliffe Hospital, OX3 9DU, United Kingdom

^b^ Departments of Psychiatry, Neurology, and Epidemiology and Biostatistics, University of California San Francisco, 4150 Clement Street, San Francisco, CA 94121, USA

^c^ Department of Anatomy and Neurosciences, VU University Medical Center, 1007 MC, Amsterdam, The Netherlands

^d^ Global Brain Health Institute, Memory and Aging Center, Department of Neurology, University of California San Francisco, CA, 675 Nelson Rising Lane, San Francisco, CA 94143, USA

^e^ Wellcome Centre for Integrative Neuroscience, Oxford Centre for Human Brain Activity, Department of Psychiatry, University of Oxford, John Radcliffe Hospital, OX3 9DU, United Kingdom

Correspondence to be addressed to: [claire.sexton@gbhi.org](mailto:claire.sexton@gbhi.org)

**Alternative methods for white matter imaging**

DTI is just one of a number of methods for imaging WM. While an extensive discussion of all possible methods is outside of the scope of this review (but see (Alexander et al., 2017; Ghosh and Deriche, 2016; Jelescu and Budde, 2017; Novikov et al., 2018b, 2018a; Sampaio-Baptista and Johansen-Berg, 2017; Tournier et al., 2011; Winston, 2012; Wozniak and Lim, 2006), here, we provide a brief overview of WM imaging methods, categorised into (1) methods that use diffusion MRI to map WM microstructure, (2) methods that use diffusion MRI to examine WM macrostructure (e.g. WM lesions), and (3) alternative MR methods to explore WM characteristics.

Amongst the methods that aim to map WM microstructure, two approaches can be distinguished: (1) signal representations, and (2) tissue models (also known as compartment models). The signal representations aim to describe the diffusion signal in a voxel without explicitly modelling the underlying tissue microstructure. Diffusion tensor imaging (DTI) is an example of such a signal model. Other signal models include q-space imaging (Cohen and Assaf, 2002), in which a generic diffusion probability density function is estimated, generalized diffusion tensor imaging (GDT) (Liu et al., 2010), diffusion kurtosis imaging (DKI) (Jensen et al., 2005), diffusion spectrum imaging (DSI) (Wedeen et al., 2005), q-ball imaging (Tuch et al., 2003), and Mean Apparent Propagator (MAP) (Özarslan et al., 2013). While these representations can be applied to any tissue type and are highly sensitive to WM changes, the parameters often lack specificity towards WM tissue microstructure. Specifically, while the diffusion tensor model has proven to be a useful and simple approach for characterizing WM bundles, it does not accurately reflect WM tissue properties, such as myelination, axon diameter or fibre density, and is known to perform poorly in regions with crossing fibers.

The tissue models, on the other hand, model the tissue microstructure explicitly by creating compartments that are assumed to correspond to cellular features (e.g. a cylinder representing axons). For instance, the ball-and-stick model comprises two compartments: an intra-axonal part modelled as stick, and extra-axonal part modelled as ball (i.e. isotropic diffusion), together allowing the fiber orientation and volume fraction of both components to be determined (Behrens et al., 2003). More recent models include the composite hindered and restricted model of diffusion (CHARMED)(Assaf and Basser, 2005), its extension AxCaliber (Assaf et al., 2008), neurite orientation dispersion and density estimation (NODDI) (Zhang et al., 2012), Diffusion Basis Spectrum Imaging (DBSI) (Wang et al., 2011), and White Matter Tract Integrity (WMTI) (Fieremans et al., 2011) (but see (Alexander et al., 2017) for a more complete overview). Common to all these models is that they assume that tissue can be modelled by two or three compartments. However, each of these models has its own set of assumptions and constraints, and allows for different metrics to be estimated (Alexander et al., 2017; Jelescu and Budde, 2017). NODDI, for instance, has been used to quantify the density and dispersion of neurites (axons and dendrites), two factors that are thought to independently influence FA and change with ageing (Billiet et al., 2015; Miller et al., 2016). More recently, WMTI has shown that age-related change in WM tracts (in particular the late-myelinating fibers) may be more specific to the extra-axonal environment, suggesting that myelin breakdown may be driving these changes (Benitez et al., 2018).

It is important to note that the tissue models are oversimplifications of tissue microstructure and that simply applying one of the biophysical models does not necessarily improve tissue characterisation. For instance, model assumptions may not hold in certain types of pathologies, potentially resulting in misleading interpretations (Jelescu and Budde, 2017). Nevertheless, nearly all models outperform DTI in their ability to characterise tissue microstructure. The field of tissue diffusion modelling is fast moving, with new methods being developed and validation studies underway. Researchers or clinicians interested in applying such novel diffusion modelling techniques are recommended to explore the methods in the design phase of the study, as well as to consider adapting scan protocols to further optimise datasets for diffusion modelling (Alexander et al., 2017).

In addition to the diffusion MRI methods outlined above, there are several other techniques that are used to study the brain’s WM (Wozniak and Lim, 2006). Volumetric approaches have been used to examine macrostructural properties of WM, like WM volume and WM lesions. Both properties are known to change with ageing (Gunning-Dixon et al., 2009; Prins and Scheltens, 2015) and can be quantified using manual techniques and (semi)automated methods, such as voxel based morphometry (VBM) for WM volumes and novel tools like Brain Intensity AbNormality Classification Algorithm (BIANCA) (Griffanti et al., 2016) for WM hyperintensities.

Finally, other MRI-based techniques have provided insight into myelination, which cannot be done directly with diffusion MRI. T2 relaxography is one such technique that allows measurements of the myelin water fraction within tissue (Arshad et al., 2016; MacKay et al., 1994). Similarly, myelin measures can be derived with techniques such as magnetization transfer (Sampaio-Baptista and Johansen-Berg, 2017), which is based on the magnetization of macromolecules, or magnetic resonance spectroscopy, which provides a measure of the chemical composition of tissue (such as myelin) (Küker et al., 2004), and via positron emission tomography (PET) (Stankoff et al., 2011).

**References**

Alexander, D.C., Dyrby, T.B., Nilsson, M., Zhang, H., 2017. Imaging brain microstructure with diffusion MRI: Practicality and applications. NMR Biomed. e3841.

Arshad, M., Stanley, J.A., Raz, N., 2016. Adult age differences in subcortical myelin content are consistent with protracted myelination and unrelated to diffusion tensor imaging indices. Neuroimage 143, 26–39.

Assaf, Y., Basser, P.J., 2005. Composite hindered and restricted model of diffusion (CHARMED) MR imaging of the human brain. Neuroimage 27, 48–58.

Assaf, Y., Blumenfeld-Katzir, T., Yovel, Y., Basser, P.J., 2008. AxCaliber: A method for measuring axon diameter distribution from diffusion MRI. Magn. Reson. Med. 59, 1347–1354.

Behrens, T.E.J., Woolrich, M.W., Jenkinson, M., Johansen-Berg, H., Nunes, R.G., Clare, S., Matthews, P.M., Brady, J.M., Smith, S.M., 2003. Characterization and Propagation of Uncertainty in Diffusion-Weighted MR Imaging. Magn. Reson. Med. 50, 1077–1088.

Benitez, A., Jensen, J.H., Falangola, M.F., Nietert, P.J., Helpern, J.A., 2018. Modeling white matter tract integrity in aging with diffusional kurtosis imaging. Neurobiol. Aging 70, 265–275.

Billiet, T., Vandenbulcke, M., Mädler, B., Peeters, R., Dhollander, T., Zhang, H., Deprez, S., Van den Bergh, B.R.H., Sunaert, S., Emsell, L., 2015. Age-related microstructural differences quantified using myelin water imaging and advanced diffusion MRI. Neurobiol. Aging 36, 2107–2121.

Cohen, Y., Assaf, Y., 2002. High b-value q-space analyzed diffusion-weighted MRS and MRI in neuronal tissues - A technical review. NMR Biomed. 15, 516–542.

Fieremans, E., Jensen, J.H., Helpern, J.A., 2011. White matter characterization with diffusional kurtosis imaging. Neuroimage 58, 177–188.

Ghosh, A., Deriche, R., 2016. A survey of current trends in diffusion MRI for structural brain connectivity. J. Neural Eng. 13, 011001.

Griffanti, L., Zamboni, G., Khan, A., Li, L., Bonifacio, G., Sundaresan, V., Schulz, U.G., Kuker, W., Battaglini, M., Rothwell, P.M., Jenkinson, M., 2016. BIANCA (Brain Intensity AbNormality Classification Algorithm): A new tool for automated segmentation of white matter hyperintensities. Neuroimage 141, 191–205.

Gunning-Dixon, F.M., Brickman, A.M., Cheng, J.C., Alexopoulos, G.S., 2009. Aging of cerebral white matter: a review of MRI findings. Int J Geriatr Psychiatry 2009; 24, 109–117.

Jelescu, I.O., Budde, M.D., 2017. Design and Validation of Diffusion MRI Models of White Matter. Front. Phys. 5, 61.

Jensen, J.H., Helpern, J.A., Ramani, A., Lu, H., Kaczynski, K., 2005. Diffusional kurtosis imaging: The quantification of non-Gaussian water diffusion by means of magnetic resonance imaging. Magn. Reson. Med. 53, 1432–1440.

Küker, W., Ruff, J., Gaertner, S., Mehnert, F., Mader, I., Nägele, T., 2004. Modern MRI tools for the characterization of acute demyelinating lesions: value of chemical shift and diffusion-weighted imaging. Neuroradiology 46, 421–426.

Liu, C., Mang, S.C., Moseley, M.E., 2010. In vivo generalized diffusion tensor imaging (GDTI) using higher-order tensors (HOT). Magn. Reson. Med. 63, 243–252.

MacKay, A., Whittall, K., Adler, J., Li, D., Paty, D., Graeb, D., 1994. In vivo visualization of myelin water in brain by magnetic resonance. Magn. Reson. Med. 31, 673–677.

Miller, K.L., Alfaro-Almagro, F., Bangerter, N.K., Thomas, D.L., Yacoub, E., Xu, J., Bartsch, A.J., Jbabdi, S., Sotiropoulos, S.N., Andersson, J.L.R., Griffanti, L., Douaud, G., Okell, T.W., Weale, P., Dragonu, I., Garratt, S., Hudson, S., Collins, R., Jenkinson, M., Matthews, P.M., Smith, S.M., 2016. Multimodal population brain imaging in the UK Biobank prospective epidemiological study. Nat. Neurosci. 19, 1523–1536.

Novikov, D.S., Fieremans, E., Jespersen, S.N., Kiselev, V.G., 2018a. Quantifying brain microstructure with diffusion MRI: Theory and parameter estimation. NMR Biomed. 1–53.

Novikov, D.S., Kiselev, V.G., Jespersen, S.N., 2018b. On modeling. Magn. Reson. Med. 79, 3172–3193.

Özarslan, E., Koay, C.G., Shepherd, T.M., Komlosh, M.E., İrfanoğlu, M.O., Pierpaoli, C., Basser, P.J., 2013. Mean apparent propagator (MAP) MRI: A novel diffusion imaging method for mapping tissue microstructure. Neuroimage 78, 16–32.

Prins, N.D., Scheltens, P., 2015. White matter hyperintensities, cognitive impairment and dementia: an update. Nat. Rev. Neurol. 11, 157–165.

Sampaio-Baptista, C., Johansen-Berg, H., 2017. White Matter Plasticity in the Adult Brain. Neuron 96, 1239–1251.

Stankoff, B., Freeman, L., Aigrot, M.S., Chardain, A., Dollé, F., Williams, A., Galanaud, D., Armand, L., Lehericy, S., Lubetzki, C., Zalc, B., Bottlaender, M., 2011. Imaging central nervous system myelin by positron emission tomography in multiple sclerosis using [methyl-11C]-2-(4-methylaminophenyl)- 6-hydroxybenzothiazole. Ann. Neurol. 69, 673–680.

Tournier, J.-D.J., Mori, S., Leemans, A., 2011. Diffusion tensor imaging and beyond. Magn. Reson. Med. 65, 1532–56.

Tuch, D.S., Reese, T.G., Wiegell, M.R., Wedeen, V.J., 2003. Diffusion MRI of Complex Neural Architecture. Neuron 40, 885–895.

Wang, Y., Wang, Q., Haldar, J.P., Yeh, F.C., Xie, M., Sun, P., Tu, T.W., Trinkaus, K., Klein, R.S., Cross, A.H., Song, S.K., 2011. Quantification of increased cellularity during inflammatory demyelination. Brain 134, 3587–3598.

Wedeen, V.J., Hagmann, P., Tseng, W.Y.I., Reese, T.G., Weisskoff, R.M., 2005. Mapping complex tissue architecture with diffusion spectrum magnetic resonance imaging. Magn. Reson. Med. 54, 1377–1386.

Winston, G.P., 2012. The physical and biological basis of quantitative parameters derived from diffusion MRI. Quant. Imaging Med. Surg. 2, 254–65.

Wozniak, J.R., Lim, K.O., 2006. Advances in white matter imaging: A review of in vivo magnetic resonance methodologies and their applicability to the study of development and aging. Neurosci. Biobehav. Rev. 30, 762–774.

Zhang, H., Schneider, T., Wheeler-Kingshott, C.A., Alexander, D.C., 2012. NODDI: Practical in vivo neurite orientation dispersion and density imaging of the human brain. Neuroimage 61, 1000–1016.
